# Biological Control of Mango Dieback Disease Caused by *Lasiodiplodia theobromae* Using Streptomycete and Non-streptomycete Actinobacteria in the United Arab Emirates

**DOI:** 10.3389/fmicb.2018.00829

**Published:** 2018-05-04

**Authors:** Fatima H. Kamil, Esam E. Saeed, Khaled A. El-Tarabily, Synan F. AbuQamar

**Affiliations:** ^1^Department of Biology, United Arab Emirates University, Al-Ain, United Arab Emirates; ^2^School of Veterinary and Life Sciences, Murdoch University, Murdoch, WA, Australia

**Keywords:** actinobacteria, antibiosis, biocontrol, chitinase, dieback, mango, *Lasiodiplodia theobromae*, UAE

## Abstract

Dieback caused by the fungus *Lasiodiplodia theobromae* is an important disease on mango plantations in the United Arab Emirates (UAE). In this study, 53 actinobacterial isolates were obtained from mango rhizosphere soil in the UAE, of which 35 (66%) were classified as streptomycetes (SA) and 18 (34%) as non-streptomycetes (NSA). Among these isolates, 19 (12 SA and 7 NSA) showed antagonistic activities against *L. theobromae* associated with either the production of diffusible antifungal metabolites, extracellular cell-wall-degrading enzymes (CWDEs), or both. Using a “novel” mango fruit bioassay, all isolates were screened *in vivo* for their abilities to reduce lesion severity on fruits inoculated with *L. theobromae*. Three isolates, two belonging to *Streptomyces* and one to *Micromonospora* spp., showed the strongest inhibitory effect against this pathogen *in vitro* and were therefore selected for tests on mango seedlings. Our results revealed that the antifungal action of *S. samsunensis* UAE1 was related to antibiosis, and the production of CWDEs (i.e., chitinase) and siderophores; whilst *S. cavourensis* UAE1 and *M. tulbaghiae* UAE1 were considered to be associated with antibiotic- and CWDE-production, respectively. Pre-inoculation in greenhouse experiments with the most promising actinobacterial isolates resulted in very high levels of disease protection in mango seedlings subsequently inoculated with the pathogen. This was evident by the dramatic reduction in the estimated disease severity indices of the mango dieback of individual biocontrol agent (BCA) applications compared with the pathogen alone, confirming their potential in the management of mango dieback disease. *L. theobromae*-infected mango seedlings treated with *S. samsunensis* showed significantly reduced number of defoliated leaves and conidia counts of *L. theobromae* by 2- and 4-fold, respectively, in comparison to the other two BCA applications. This indicates that the synergistic antifungal effects of *S. samsunensis* using multiple modes of action retarded the *in planta* invasion of *L. theobromae*. This is the first report of BCA effects against *L. theobromae* on mango seedlings by microbial antagonists. It is also the first report of actinobacteria naturally existing in the soils of the UAE or elsewhere that show the ability to suppress the mango dieback disease.

## Introduction

Mango (*Mangifera indica* L.), frequently recognized as “the king of fruits,” is a popular fruit in tropical and subtropical regions (Usman et al., 2001; Berardini et al., 2005). Due to its delicious taste, high nutritional value and economical importance in international markets, mango has increasingly been cultivated not only in its traditional producing areas, but also in non-traditional production countries such as the United Arab Emirates (UAE) (Nelson, 2008; Saeed et al., 2017a). Mango can be attacked by a number of bacterial and fungal pathogens causing several diseases in all parts of the tree and at all stages of its life (Ploetz, 2004). Studies have identified the pathogenic fungus, *Lasiodiplodia theobromae* (Pat.) Griffon and Maubl. (Zambettakis, 1954; Sutton, 1980), as the causal agent of mango dieback disease in different areas of the world, including Brazil, Korea, India, Oman, Pakistan, USA (Sharma et al., 1994; Ploetz et al., 1996; Al Adawi et al., 2003; Khanzada et al., 2004; de Oliveira Costa et al., 2010; Hong et al., 2012) and the UAE (Saeed et al., 2017a). Thus, dieback is considered to be the most destructive disease, leading to significant yield loss and low fruit quality of mango (Ploetz, 2003).

The disease symptoms of dieback on mango are commonly associated with drying and withering of twigs from top downwards, followed by discoloration, drying and eventual dropping of leaves (Khanzada et al., 2004). Other symptoms can also be observed on other parts of the tree, including reproductive structures (Naqvi et al., 2014). In advanced stages of the disease, branches dry one after another, resulting in the appearance of bare twigs and the decline of trees. Typically, a complete wilting and death of the affected mango trees may occur within weeks or few months after infestation with *L. theobromae* (Saeed et al., 2017a). Regrettably, once the symptoms of dieback are present, it is very hard to save the mango orchard or reverse the disease development. In the field, poor orchard management and unfavorable environmental stresses such as drought, heat, sun scorch, water stress, salinity and nutritional deficiency, can also provoke the progress of disease (Kazmi et al., 2005; Paolinelli-Alfonso et al., 2016). Studies have shown that most common varieties of mango are highly susceptible to dieback disease caused by *L. theobromae* (Ramos et al., 1997). In general, dieback is a serious disease of mango, which causes damage to tree health and considerable loss of fruit yield. Thus, there is an urgent need for research to find innovative and safe solutions for this destructive disease.

Unfortunately, strategies involving early applications with chemical fungicides on affected plants are still the main means to lessen the severity of most diseases on crops (Saeed et al., 2016). The increasing awareness of fungicide-related risks have further highlighted the need for adopting alternative and sustainable methods, such as proper horticultural practices, biological control (or biocontrol) agents (BCAs) and safe natural compounds, to replace the dependence on chemical fungicides for disease control (Ma and Michailides, 2005). Integrated pest management (IPM) aims at using a combination of practices and minimizing chemical inputs that are only applied when needed (López-Escudero and Mercado-Blanco, 2011). In that regard, efforts toward using natural enemies native to the same environment can effectively reduce or exterminate pathogen populations (AbuQamar et al., 2017; Syed Ab Rahman et al., 2018). *In vitro* and field studies were previously applied against *L. theobromae* using species of *Bacillus* to control seed and seedling rot of bottle gourd (Sultana and Ghaffar, 2010), *Trichoderma* and *Aspergillus* to control inflorescence blight of cashew (Adeniyi et al., 2013), and *Pseudomonas* to control stem-end rot on mango fruits (Seethapathy et al., 2016). No information exists on potential antagonistic microorganisms that have been identified to be capable of managing mango dieback disease under greenhouse/field conditions caused by *L. theobromae*, as a component of an IPM strategy.

Actinobacteria are a group of Gram-positive bacteria that include some of the most common soil bacteria (Locci and Sharples, 1984). *Streptomyces*, a common actinobacterial genus, is also a biologically active “vehicle” of the soil microbiota (Barka et al., 2016) that can play a vital role against phytopathogens including fungi and oomycetes (El-Tarabily et al., 2009; Saeed et al., 2017b). In comparison, other genera of actinobacteria, such as *Actinoplanes, Microbispora, Micromonospora* and *Streptosporangium* have rarely been investigated as BCAs and/or plant growth promoters (PGPs) (El-Tarabily et al., 1997, 2009; El-Tarabily and Sivasithamparam, 2006).

The biological control of soil-borne fungal pathogens by actinobacteria may involve several mechanisms (Doumbou et al., 2001; Whipps, 2001; Kinkel et al., 2012), including inhibition of pathogen growth through the production of antifungal metabolites (Getha et al., 2005; Palaniyandi et al., 2013; Saeed et al., 2017b), production of siderophores (Xue et al., 2013), destructive parasitism (El-Tarabily et al., 1997), competition for infection sites and/or nutrient resources (Cook and Baker, 1983), and lysis of fungal hyphae by the production of cell-wall-degrading enzymes (CWDEs; glucanases and chitinases) (Valois et al., 1996; Mahadevan and Crawford, 1997; El-Tarabily et al., 2000; El-Tarabily, 2006; Singh and Gaur, 2016). Soil-borne beneficial bacteria, such as actinobacteria, may also trigger induced systemic resistance (ISR) in plants and reduce the effects of pathogen attacks through the induction of their defense mechanisms (Martínez-Hidalgo et al., 2015). As a result, application of BCAs represents an environmentally-friendly strategy for sustainable agriculture and cost-efficient protection that can enhance crop productivity.

Previously, we identified *L. theobromae* as the mango dieback pathogen in the UAE, and evaluated the activity of systemic fungicide treatments in providing protection against this disease on mango plants in the greenhouse and in the field (Saeed et al., 2017a). In the current study, we specifically targeted actinobacteria as potential candidates as they are mostly well-adapted to be active in the arid environment, in comparison to other bacteria and fungi (Goodfellow and Williams, 1983). We isolated a wide spectrum of streptomycete actinobacteria (SA) and non-streptomycete actinobacteria (NSA) from mango rhizosphere. The objectives of the present investigation were to: (i) screen all the actinobacterial isolates for their ability to produce *in vitro*, diffusible antifungal metabolite(s) and/or CWDEs capable of inhibiting *L. theobromae*, (ii) select the promising isolates using a novel mango fruit bioassay which we developed, to assess their potential to reduce disease progression on fruits, and (iii) evaluate the differences in the potential of the promising isolates with known mode(s) of antagonism under greenhouse conditions, for their effectiveness in controlling mango dieback disease. Our results demonstrate the potential to use selected actinobacteria with more than a single mode of action as antagonists to be incorporated into sustainable IPM strategies to manage dieback disease in mango orchards in the UAE and elsewhere.

## Materials and methods

### Fungal growth and culture

The pathogen, *L. theobromae* (DSM 105134), was previously reported as the cause of mango dieback disease in the UAE (Saeed et al., 2017a). Strain DSM 105134 was isolated from tissues sampled from diseased mango trees affected by *L. theobromae*, and identified using combined *ITS* sequences (GenBank accession number: MF114110). The pathogen was cultured on potato dextrose agar (PDA, pH 6.0; Lab M Limited, Lancashire, United Kingdom) plates, supplemented with 25 mg l^−1^ ampicillin (Sigma–Aldrich Chemie GmbH, Taufkirchen, Germany) to suppress bacterial contaminants. The fungus was sub-cultured every 10 days on PDA plates at 28°C.

### Isolation of SA and NSA from mango rhizosphere

Five rhizosphere soil samples were collected from 30 cm depth under healthy mango trees in sealable plastic bags. The rhizosphere soil samples were air-dried for 4 days at 25°C (Williams et al., 1972), passed through a 5 mm mesh sieve, and stored prior further analyses.

The soil dilution plate method was used to isolate SA from each rhizosphere sample using inorganic salt starch agar (Küster, 1959) supplemented with the antifungal antibiotics nystatin and cycloheximide (50 μg ml^−1^ each; Sigma-Aldrich). In order to increase and decrease the populations of filamentous actinobacteria and other bacteria, respectively, the soil pre-treatments described by Hayakawa and Nonomura (1987) was used. For each dilution, seven plates were used and incubated at 28°C in dark for 7 days.

For the NSA recovery, four polyvalent *Streptomyces* phages (El-Tarabily, 2006) were used to reduce the dominance of SA on inorganic salt starch agar plates (Kurtböke et al., 1992). The stock phage suspension (10^12^ plaque forming units ml^−1^) was prepared by mixing high-titer phage suspensions of each polyvalent *Streptomyces* phage. Seven plates were inoculated with 0.2 ml aliquots of the phage-treated soil suspension, dried and incubated in dark at 28°C for 14 days. Control treatments were considered as plates without phages.

Colonies of SA and NSA, expressed as log_10_ colony forming units (cfu) g^−1^ dry soil, were purified on oatmeal agar plates (ISP medium 3) amended with 0.1% yeast extract (Küster, 1959). Colonies were identified based on morphological features, distribution of aerial/substrate mycelia, presence/absence of aerial mycelia, and the stability/fragmentation of substrate mycelia (Cross, 1989).

### Detection of the antifungal and CWDE activities

We characterized all actinobacterial isolates based on their ability to secrete diffusible antifungal metabolites active against *L. theobromae* using the cut-plug method (Pridham et al., 1956). The actinobacterial isolates were inoculated on fish meal extract agar (El-Tarabily et al., 1997) plates and incubated at 28°C in dark for 7 days. PDA-seeded plates were prepared by initially cultivating *L. theobromae* on PDA slants at 28°C until sporulation, which were then flooded with 50 mM phosphate buffer (pH 6.8) (Saeed et al., 2017b). Spores and some mycelial fragments were homogenized at 4,000 rpm for 20 min; and the resulting supernatants were diluted in PDA plates. The inoculum consisted of approximately 10^8^ cfu ml^−1^. PDA-seeded plates with non-inoculated agar plugs served as control. Plugs were transferred from the actinobacterial cultures on fish meal extract agar with a sterilized 11 mm cork-borer onto PDA plates seeded with *L. theobromae* kept at 28°C in dark for 5 days. The diameters of zones of inhibition were determined. Five plates were used for each actinobacterial isolate. The most promising antifungal metabolite-producing isolates showing the largest zone of inhibition were picked for further experiments; and the remaining of the isolates were not used in the subsequent tests.

All isolates were also tested for their abilities to produce clearing zones on *L. theobromae* mycelial fragment agar as an indicator of preliminary production of CWDEs according to Valois et al. (1996). Large (>30 mm) and small (<30 mm) diameters represented high and low CWDE activities, respectively. In addition, all obtained isolates were evaluated for their potential to produce chitinase enzyme. Each isolate was inoculated onto colloidal chitin agar plates, and incubated at 28°C in dark for 7 days (Gupta et al., 1995). The clearing zones surrounding the colonies were measured and used to detect the chitinase activity. Large (>30 mm) and small (<30 mm) diameters represented high and low chitinase activities, respectively. Five replicate plates were used for each actinobacterial isolate. The most promising, highly active CWDE-producing isolates showing the largest clearing zones on both mycelial fragment agar and colloidal chitin agar plates were chosen for further experiments.

### Mango fruit bioassay

A novel mango fruit bioassay was developed in our laboratory to determine the ability of the most promising candidates to suppress or reduce disease development (lesion formation) following inoculation with *L. theobromae in vivo*. The mango fruit bioassay was modified according to previous tests of carrot bioassay against *Pythium coloratum* (El-Tarabily et al., 1997) and mango fruit pathogenicity against *L. theobromae* (Saeed et al., 2017a).

Mature mango fruits (cv. Badami) were placed in plastic trays on sterile paper towels moistened with sterile distilled water, and were inoculated by placing the agar plugs (11 mm) colonized by the actinobacterial isolates and/or *L. theobromae*, described above, onto each mango fruit according to the following combinations: (i) a sterile non-inoculated PDA agar plug (control; C); (ii) the antagonist alone (BCA) with a sterile PDA agar plug above it; (iii) *L. theobromae* (*Lt*) alone with a sterile PDA agar plug below it; and (iv) pairing *L. theobromae* and the antagonists together (BCA+*Lt*), with the BCA on the mango surface and *L. theobromae*-inoculated plug on top of the BCA. The antagonists were inoculated onto the mango surface 24 h prior the pathogen in order to allow time for the secretion of antifungal metabolites and/or chitinase onto the mango surface. Each mango fruit was inoculated with the four treatment combinations for each BCA of four fruits/tray in triplicates. Trays were covered with aluminum foil and incubated under humid conditions in dark at 28°C for 4 days. The lesion diameters were measured in order to determine disease indices. To fulfill Koch's postulates, all diseased fruit tissues were incubated on PDA plates at 28°C in dark for 5 days.

### Assays of producing diffusible antifungal metabolites or chitinase

We assessed the three most promising antagonistic BCAs for their ability to secrete diffusible antifungal metabolites active against *L. theobromae* using the cup plate technique as previously described (Bacharach and Cuthbertson, 1948). Inocula for the preparation of the *L. theobromae*-seeded PDA plates were prepared, as described above, for the cut-plug method. In order to assess the inhibition of *L. theobromae* by the diffusible antifungal metabolites on fish meal extract agar (El-Tarabily et al., 1997) or by the chitinase on colloidal chitin agar (El-Tarabily et al., 2000), a dialysis membrane overlay technique (Gibbs, 1967) was used.

Briefly, dialysis membrane (Type 45311; Union Carbide Corporation, IL, USA) with adhering colonies were removed from the agar plates and the center of each plate was inoculated with a disc (5 mm diameter) of *L. theobromae* culture. At the end of the incubation period, the colony diameter of *L. theobromae* was measured. The agar plugs were further transferred to a fresh PDA plate and incubated at 28°C for 5 days to determine whether the diffused metabolites/chitinase were fungistatic (pathogen growth from the plug) or fungicidal (no pathogen growth from the plug).

### Volatile antifungal compounds, hydrocyanic acid and siderophore production

Production of volatile antifungal compounds (Payne et al., 2000) by the BCAs was examined using fish meal extract agar. For the production of hydrogen cyanide (hydrocyanic acid), the BCAs were inoculated on tryptic soy agar medium (Lab M Limited) supplemented with 4.4 g glycine l^−1^. The plates were inverted and a piece of filter paper (soaked in 0.5% picric acid in 2% sodium carbonate) was placed in the lid of each Petri dish, and incubated at 28°C for 5 days (Bakker and Schippers, 1987). Discoloration of the filter paper to orange brown after incubation indicates production of hydrogen cyanide (Castric, 1975).

For siderophore production, plates of chrome azurol S (CAS) agar developed by Schwyn and Neilands (1987), were inoculated with the BCAs and incubated at 28°C in dark for 7 days. Development of yellow-orange halo zone around the colony was considered positive for siderophore production.

### Determination of CWDE activities of the BCA candidates

Erlenmeyer flasks containing 50 ml of minimal synthetic medium (Tweddell et al., 1994) supplemented with 2 mg ml^−1^ of either *L. theobromae* cell wall fragments, colloidal chitin, or laminarin (Sigma-Aldrich) were prepared. Flasks containing each substrate were inoculated with 2 ml of a 20% glycerol suspension of each BCA (10^8^ cfu ml^−1^), incubated on a rotary shaker (Model G76, New Brunswick Scientific, NJ, USA) at 250 rpm for 7 days, and further centrifuged at 12,000 × *g* for 30 min. The supernatant was filtered using 0.22 μm Millipore membranes (Millipore Corporation, MA, USA) and used as a source of crude enzymes (El-Tarabily, 2003).

Chitinase and ß-1,3-glucanase activities were determined by measuring the release of N-acetyl-D-glucosamine and the amount of reducing sugars liberated using dinitrosalicylic acid solution (Miller, 1959), respectively. The protein content of the enzyme solution was determined as described by Lowry et al. (1951) using Folin phenol reagent.

### Effect of BCA crude culture filtrates on mycelia and conidia of *L. theobromae*

The filter-sterilized crude culture filtrate for each BCA (section Determination of CWDE Activities of the BCA Candidates) using fish meal extract broth or colloidal chitin broth (Gupta et al., 1995) was proportionally poured in PDA plates. The medium was inoculated with a 5 mm diameter agar plug colonized with *L. theobromae* mycelium (placed upside down). The colony diameter (mm) of *L. theobromae* was measured after 5 days at 28°C.

The crude culture filtrate prepared from fish meal extract broth or colloidal chitin broth was also proportionally mixed with potato dextrose broth (PDB; Lab M) (Lorito et al., 1993). The PDB was inoculated with a 5 mm diameter agar plug colonized with *L. theobromae*. The dry weight of *L. theobromae* was measured after 10 days of incubation in dark at 28°C.

The effect of the crude culture filtrate of each BCA on mature conidia germination and germ tube elongation of *L. theobromae* was carried out in PDB according to Lorito et al. (1993). The percentage spore germination and average germ tubes lengths were microscopically determined after 24 h at 40X using Nikon-Eclipse 50i light microscope (Nikon Instrument Inc., NY, USA) and compared with the control (non-inoculated filter-sterilized fish meal extract broth or colloidal chitin broth).

The effect of the crude culture filtrate of the three BCAs on the morphology of *L. theobromae* hyphae was assessed (Sneh, 1981). At sampling, *L. theobromae* hyphae treated with the BCA was microscopically examined at 100X using a light microscope. *L. theobromae* mycelium incorporated with non-inoculated filter-sterilized fish meal extract broth or colloidal chitin broth served as control treatments. Three replicates were used at each sampling.

### Identification and phylogenetic analysis of the BCA candidates

The identification of the three promising BCAs, BCA1 (isolate #12), BCA2 (isolate #29) and BCA3 (isolate #44), was carried out using 16*S* rRNA gene sequence analysis done by the Deutsche Sammlung von Mikroorganismen und Zellkulturen GmbH, (DSMZ), Braunschweig, Germany, using the primers 900R (5′-CCGTCAATTCATTTGAGTTT-3′); 357F (5′-TACGGGAGGCAGCAG-3′) and 800F (5′-ATTAGATACCCTGGTAG-3′) (Rainey et al., 1996). Sequences for BCA1, BCA2 and BCA3 were deposited in Genbank with accession numbers MG548382, MG461691, and MF872601; respectively. Phylogenetic tree was constructed to predict the species level characterization of the studied isolates using the maximum likelihood method implemented in Molecular Evolutionary Genetics Analysis 7.0 (MEGA7) software (Felsenstein, 1981; Kumar et al., 2016) after multiple alignments of the data by CLUSTAL_X (Thompson et al., 1997). In each case, bootstrap values were calculated based on 1,000 resamplings.

Identification of BCA1 and BCA2 isolates was further confirmed based on cultural, morphological, and physiological characteristics as described by Locci (1989). Scanning electron microscopy (SEM) was carried out for the three BCA isolates (BCA1, BCA2, and BCA3) using Philips XL-30 SEM (FEI Co., Eindhoven, The Netherlands) to determine the morphology of the spore chains and surface.

### Disease assays and greenhouse trials

For disease assays on mango seedlings under greenhouse conditions, the growing tip region of the stem of 12-month-old mango (cv. Badami) seedlings were surface-sterilized with 70% ethanol, mechanically wounded, and inoculated with 5 mm PDA plugs colonized with *L. theobromae* culture or non-colonized plugs (controls) (Saeed et al., 2017a). The area of inoculation was covered with Parafilm. Inoculated seedlings were kept at 28°C under greenhouse conditions, and monitored for disease development.

*In vivo* evaluations of the BCAs were also carried out on mango seedlings. We aimed to investigate the efficacy of the three BCA treatments to manage dieback disease. Similar to the *L. theobromae* treatment described above, methods of inoculation with the pathogen and BCA application were used. All BCA treatments were preventive (seedlings treated with each BCA 1 week before *L. theobromae* inoculation). The treatments/groups used for this experiment were as follows:

Healthy controls (C): Non inoculated control seedlings;

Diseased controls (*Lt*): Seedlings inoculated with *L. theobromae* only;

Biocontrol treatment without pathogen (*Ss, Sc*, or *Mt*): Seedlings inoculated with either *S. samsunensis, S. cavourensis* or *M. tulbaghiae*, respectively;

Preventive biocontrol treatment (*Ss*+*Lt*; *Sc*+*Lt*, or *Mt*+*Lt*): Seedlings inoculated with either *S. samsunensis, S. cavourensis* or *M. tulbaghiae*, respectively, 1 week before *L. theobromae* inoculation.

For each treatment/group, six plants in separate pots, arranged in a completely randomized design, were used. Control (healthy and diseased) and inoculated seedlings were maintained under controlled greenhouse conditions of 15 h light/9 h dark under fluorescent lights (160 W mol^−1^ m^−2^ s^−1^) at 28°C. Corresponding with the disease symptoms/recovery, disease severity index (DSI) was recorded at 3 and 9 weeks post inoculation (wpi) using the following scale: 0 = no apparent symptoms, 1 = 1–10%, 2 = 11–25%, 3 = 26–50%, 4 = 51–75%, and 5 = 76–100% necrotic or dark brown area around the point of inoculation (Saeed et al., 2017a). The number of falling leaves and the total number of fungal conidia in inoculated plants were determined at 6 and 9 wpi, respectively. Harvested conidia from three leaf bases of 6 inoculated seedlings per treatment were counted using a haemocytometer (Agar Scientific Limited, Essex, UK) (Saeed et al., 2017a).

### Statistical analyses

For mango fruit bioassay, the effect of actinobacteria on lesion formation was evaluated and analyzed using Analysis of Variance (ANOVA). Each mango fruit was inoculated with the four treatments and each tray contained four fruits with three replicate trays for each isolate. Significant differences between means at *P* = 0.05 were determined by Duncan's multiple range test.

ANOVA and Duncan's multiple range test at 5% level of significance were used to analyze the *in vitro* evaluation of BCA against *L. theobromae*. Experiments were repeated in triplicates using five plates per treatment for each time with similar results.

For the falling leaves and fungal conidia counts of the *in vivo* treatments against *L. theobromae* in the greenhouse trial, six plants were used for each treatment. ANOVA and Duncan's multiple range test were used to determine the statistical significance (*P* < 0.05). All experiments were repeated independently three times with similar results.

Three replicates for each group (6 plants each) were tested for the DSI of the *in vivo* treatments. ANOVA and Duncan's multiple range test were conducted to determine the statistical significance at *P* < 0.05. Similar results were obtained in each replicate. For all statistical analyses, SAS Software version 9 was used (SAS Institute, 2002).

## Results

### Production of diffusible antifungal metabolites and CWDEs by BCAs

**Figure 1:**
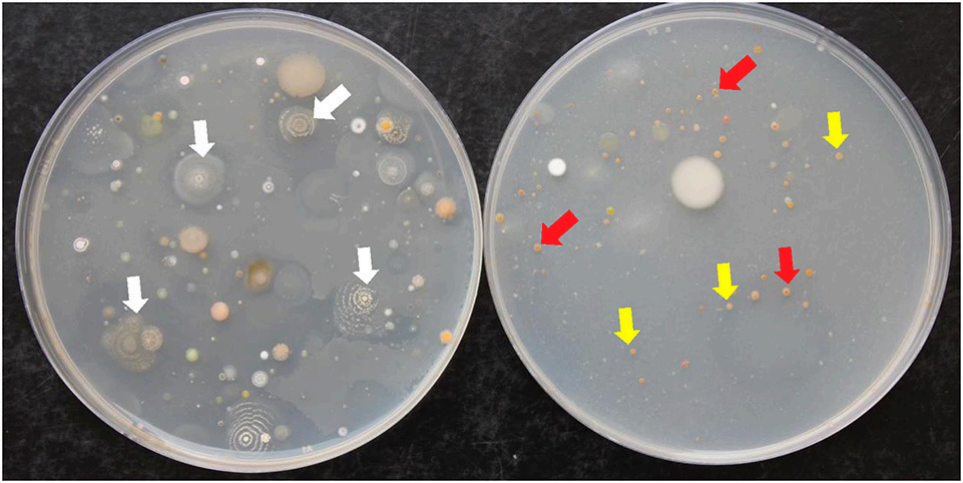
Colonies of actinobacteria isolated from mango rhizosphere grown on inorganic salt starch agar plates. Soil dilutions tubes were treated without **(Left)** and with **(Right)** four polyvalent *Streptomyces* phages. White arrows represent the dominance of streptomycete actinobacterial colonies **(Left)**; whereas yellow and red arrows represent the dominance of non-streptomycete actinobacterial colonies i.e., *Actinoplanes* and *Micromonospora* spp., respectively **(Right)**.

Fifty-three SA and NSA strains were isolated from mango rhizosphere, of which 35 SA (66.1%) and 18 NSA (33.9%) were obtained from inorganic salt starch agar plates. The *Streptomyces* phages with high polyvalency were used to facilitate the isolation of NSA from rhizosphere samples on inorganic salt starch agar plates (Figure 1). Consequently, the numbers of SA were significantly (*P* < 0.05) reduced, but the numbers of NSA increased on the plates treated with the four phages (Table 1). Therefore, SA and NSA (*Actinoplanes, Actinomadura, Microbispora, Micromonospora, Nocardia, Rhodococcus*, and *Streptosporangium* spp.) were readily isolated and identified to the genus level based on morphological features, distribution of aerial and/or substrate mycelia, presence/absence of aerial mycelia, and the stability or fragmentation of substrate mycelia.

**Table 1:** The effect of introducing four polyvalent *Streptomyces* phages on the colony-forming units of streptomycete and non-streptomycete actinobacteria from mango rhizosphere soil.

**Actinobacteria**	**Without phage**	**With phage**
	**log**_**10**_ **cfu g soil**^**−1**^	
SA	5.96 ± 0.45*a*	2.04 ± 0.31*b*
NSA	1.86 ± 0.18*a*	3.70 ± 0.26*b*

*Values are means of seven replicates ± SE. Within rows, values followed by the same letter are not significantly (P > 0.05) different according to Duncan's multiple range test. SA, streptomycete actinobacteria; NSA, non-streptomycete actinobacteria*.

**Figure 2:**
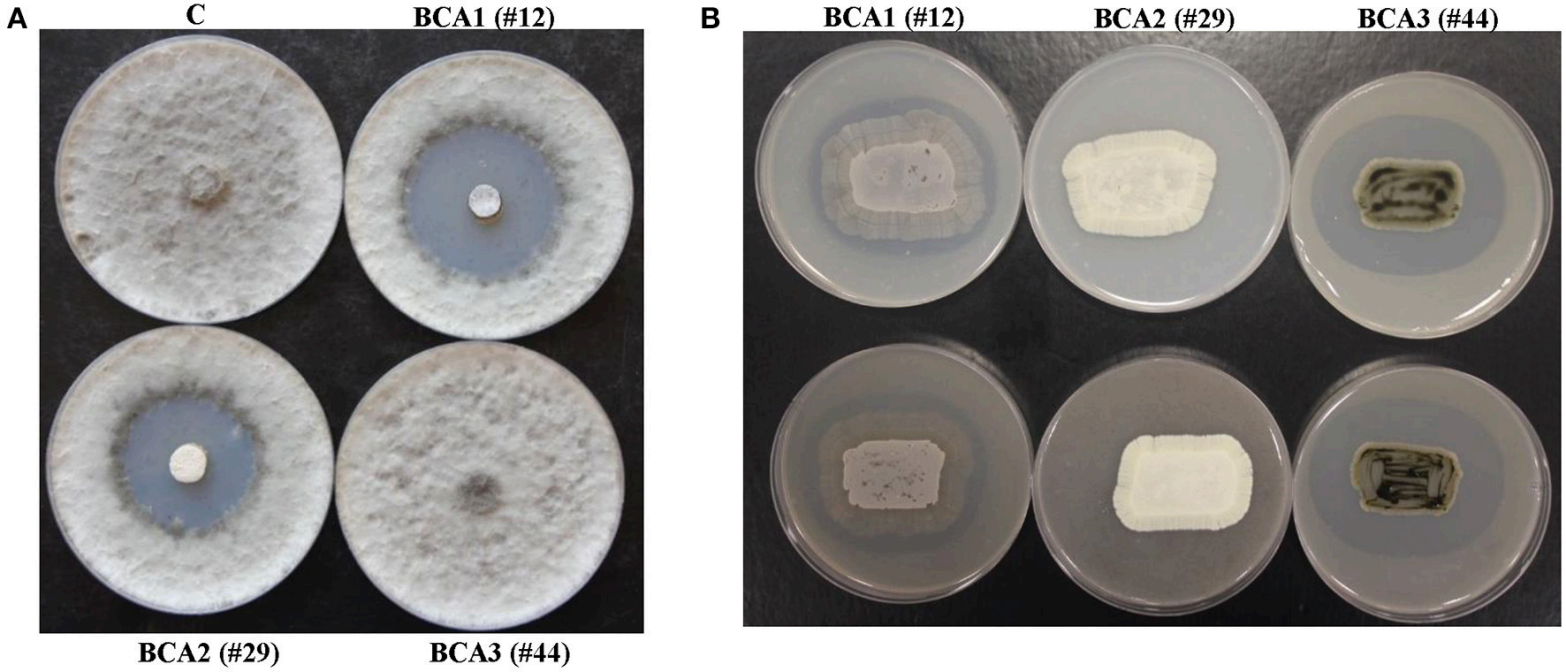
Production of diffusible antifungal metabolites and cell-wall-degrading enzymes by BCA candidates active against *Lasiodiplodia theobromae*. Inhibition of *L. theobromae* mycelial growth by the BCA1 and BCA2 using cut-plug method **(A)**; and production of chitinase enzymes by BCA1 and BCA3 grown on colloidal chitin agar (upper panel) and production of CWDEs on mycelial fragment agar (lower panel) **(B)**. In **(A)** the diffusible antifungal metabolite-producing isolate BCA1 (isolate #12; *Streptomyces samsunensis* UAE1) and BCA2 (isolate #29; *S. cavourensis* UAE1) compared to the non-diffusible antifungal metabolite-producing isolate BCA3 (isolate #44; *Micromonospora tulbaghiae* UAE1). In **(B)** production of CWDEs by BCA1 and BCA3 isolates compared to the non- CWDE producing BCA2 isolate. C, a sterile non-inoculated PDA agar plug (control).

We found that 11 out of 53 of the rhizosphere actinobacterial (7 SA and 4 NSA) isolates were capable of producing strong antifungal metabolites active against *L. theobromae* using the cut-plug method (Table 2). Eleven isolates (#3, 7, 9, 12, 16, 21, 25, 29, 42, 49, and 50) produced large zones of pathogen inhibition (>30 mm), and were considered as the most promising BCA candidates (Table 2; Figure 2A); thus were selected for further analyses. The rest of the isolates that caused very low levels of inhibition (< 30 mm) were not included in the subsequent studies.

**Table 2:** *In vitro* and *in vivo* antagonism shown by 19 isolates of streptomycete and non-streptomycete actinobacteria against *Lasiodiplodia theobromae*.

**Treatment**	**Isolate**	***In vitro***			***In vivo***
		**Diameter of inhibitionzone (mm)^a^**	**Diameter of clearingzone (mm)^b^**	**Diameter of clearingzone (mm)^c^**	**Lesion diameter(mm)^d^**
*Streptomyces* sp.	3	46.41 ± 0.49**b**	46.10 ± 0.98**d**	48.79 ± 0.35**d**	15.88 ± 1.03**b**
	7	40.40 ± 0.58**e**	0.00 ± 0.00**g**	0.00 ± 0.00**g**	32.21 ± 0.65**a**
	10	0.00 ± 0.00**i**	54.50 ± 2.16**cb**	54.96 ± 1.70**c**	33.13 ± 0.31**a**
	12 (BCA1)	49.16 ± 0.88**a**	56.04 ± 0.87**b**	57.97 ± 0.98**b**	0.00 ± 0.00**e**
	18	0.00 ± 0.00**i**	52.59 ± 0.45**c**	54.80 ± 0.65**c**	33.32 ± 0.49**a**
	21	44.40 ± 0.55**c**	0.00 ± 0.00**g**	0.00 ± 0.00**g**	32.31 ± 0.65**a**
	29 (BCA2)	42.40 ± 0.49**d**	0.00 ± 0.00**g**	0.00 ± 0.00**g**	0.00 ± 0.00**e**
	31	0.00 ± 0.00**i**	35.98 ± 0.72**f**	37.52 ± 0.55**f**	0.00 ± 0.00**e**
	41	0.00 ± 0.00**i**	40.81 ± 0.77**e**	42.06 ± 1.35**e**	33.03 ± 0.69**a**
	42	37.10 ± 0.86**f**	0.00 ± 0.00**g**	0.00 ± 0.00**g**	12.73 ± 1.74**c**
	49	31.73 ± 0.95**h**	41.72 ± 0.95**e**	43.98 ± 0.79**e**	0.00 ± 0.00**e**
	51	0.00 ± 0.00**i**	56.22 ± 1.05**b**	57.29 ± 0.53**b**	32.38 ± 0.42**a**
*Actinoplanes* sp.	9	31.23 ± 0.76**h**	0.00 ± 0.00**g**	0.00 ± 0.00**g**	0.00 ± 0.00**e**
*Microbispora* sp.	16	49.60 ± 0.60**a**	0.00 ± 0.00**g**	0.00 ± 0.00**g**	32.81 ± 0.73**a**
*Micromonospora* sp.	25	47.13 ± 0.84**b**	0.00 ± 0.00**g**	0.00 ± 0.00**g**	7.45 ± 0.57**d**
	44 (BCA3)	0.00 ± 0.00**i**	63.90 ± 1.43**a**	65.62 ± 1.30**a**	0.00 ± 0.00**e**
*Nocardia* sp.	33	0.00 ± 0.00**i**	38.18 ± 0.40**f**	39.66 ± 0.37**f**	31.90 ± 0.62**a**
*Rhodococcus* sp.	45	0.00 ± 0.00**i**	52.18 ± 1.04**c**	54.37 ± 1.18**c**	32.22 ± 0.98**a**
*Streptosporangium* sp.	50	34.53 ± 0.60**g**	54.10 ± 1.36**cb**	56.15 ± 1.29**bc**	12.01 ± 1.20**c**
Diseased control (*L. theobromae*)		ND	ND	ND	32.51 ± 0.57**a**
Healthy control (no*L. theobromae*)		ND	ND	ND	0.00 ± 0.00**e**

a *Production of diffusible antifungal metabolites active against L. theobromae using the cut-plug method*.

b *Production of chitinase on colloidal chitin agar*.

c *Production of cell-wall-degrading enzymes on mycelial fragment agar*.

d *Effect of the antagonistic BCA on L. theobromae using the in vivo mango fruit bioassay*.

*Values are means of three replicates ± SE for the in vitro experiments and in vivo experiments. Values within each column, followed by the same letter are not significantly (P > 0.05) different according to Duncan's multiple range test*.

*Isolates #12, #29 and #44 represent Streptomyces samsunensis UAE1 (BCA1), S. cavourensis UAE1 (BCA2) and M. tulbaghiae UAE1 (BCA3); respectively*.

*ND, not determined*.

Of the 53 isolates, 8 SA and 4 NSA (isolates #3, 10, 12, 18, 31, 33, 41, 44, 45, 49, 50, and 51) were ranked as highly active chitinase-producing isolates. These 12 isolates produced large clearing zones (>30 mm) around the colony on colloidal chitin agar plates and on *L. theobromae* mycelial fragment agar (Table 2; Figure 2B). The remainder of the isolates produced small clearing zones (< 30 mm) and were not further assessed. It is noteworthy to mention that four BCA candidates (isolates #3, 12, 49, and 50) produced both the diffusible antifungal metabolites and CWDEs (Table 2). This suggests that the isolated SA and NSA from the local mango rhizosphere soil samples may have antifungal activities of single or multiple modes of action against plant pathogens, including *L. theobromae*.

### Selection of the most promising antagonistic BCA candidates

**Figure 3:**
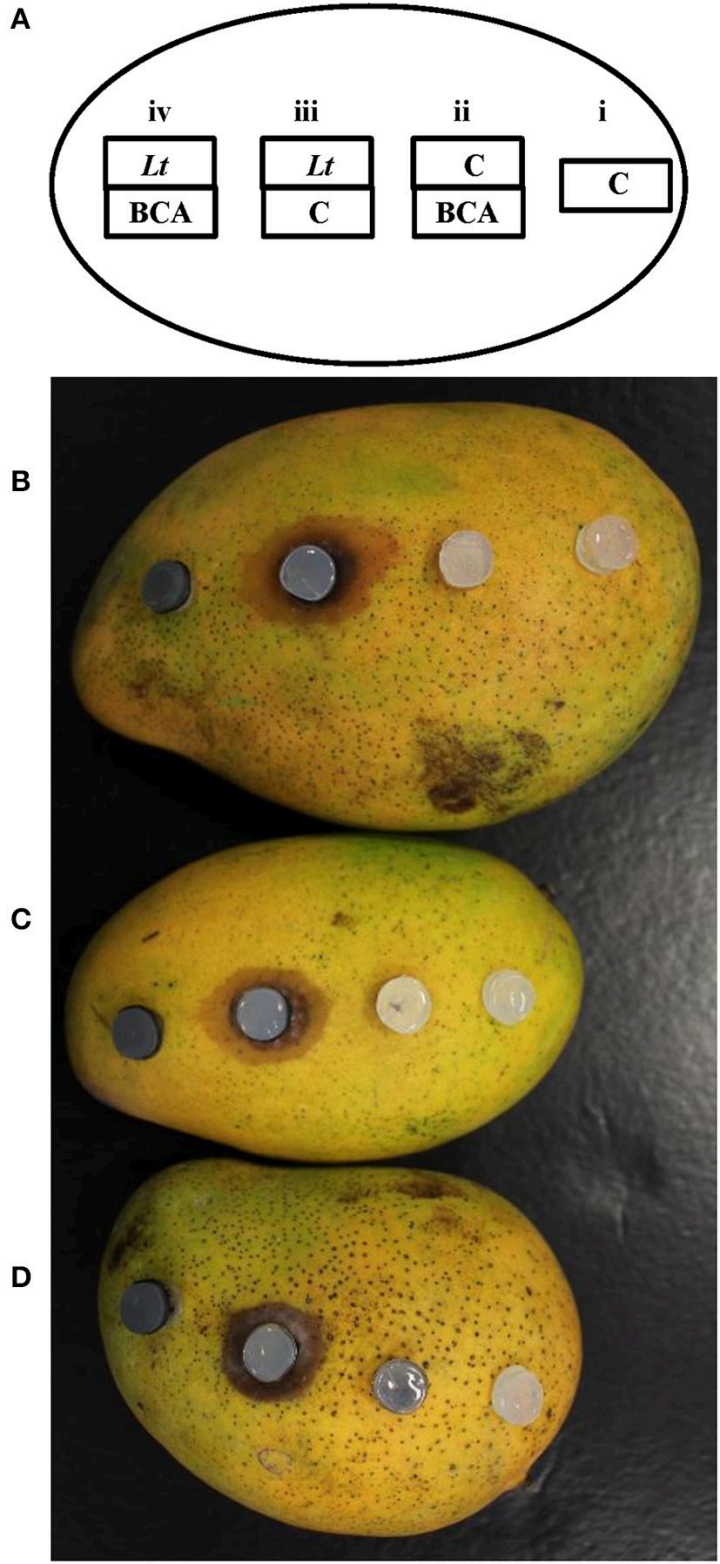
*In vivo* inhibitory effect of the BCA candidates against *Lasiodiplodia theobromae* using the “mango fruit bioassay”. An illustration showing an inoculated-mango fruit with the colonized BCA and/or *L. theobromae* agar plugs in combinations onto each mango fruit **(A)**. Mango fruit bioassays using BCA1 (isolate #12; *Streptomyces samsunensis* UAE1) **(B)**; BCA2 (isolate #29; *S. cavourensis* UAE1) **(C)**; or BCA3 (isolate #44; *Micromonospora tulbaghiae* UAE1) **(D)** as potential BCAs. (i) C, a sterile non-inoculated PDA agar plug (control); (ii) BCA, the antagonist alone with a sterile agar plug above it; (iii) *Lt, L. theobromae* inoculum alone with a sterile agar plug below it; and (iv) BCA+*Lt*, pairing *L. theobromae* and the antagonist together, with the BCA on the mango surface and *L. theobromae*-inoculated plug on top of the BCA.

We used the mango fruit bioassay method to evaluate the most effective 19 BCA candidates against *L. theobromae* (Table 2; Figure 3A). Lesions produced on the fruits by the pathogen (*Lt*) alone were relatively large, brownish, round to elliptical, water-soaked and depressed, with clear margins (Figures 3B–D). When certain isolates were paired with the pathogen (BCA+*Lt*) on the mango fruit surface, they completely suppressed the pathogen with no lesions formed compared with the treatment with the pathogen plug alone (*Lt*) (Table 2; Figure 3). Certain isolates significantly (*P* < 0.05) reduced lesion development in comparison to the treatment with the pathogen alone (Table 2).

In general, ten isolates (6 SA and 4 NSA) totally prevented or reduced lesion development to varying degrees, whilst the remaining 9 isolates failed to reduce lesion formation (Table 2). The isolates that showed production of antifungal metabolites and/or CWDEs, but failed to totally prevent lesion development on mango fruits were excluded. The mango fruit bioassay led to the selection of only three most promising antagonistic isolates: 2 SA (#12 and #29) and 1 NSA (#44) which completely prevented lesion formation on mango fruit.

Our data suggest that the three BCA candidates selected are highly effective against *L. theobromae*, and that the preventive effect of isolates #12, #29 and #44 could have the potential to manage dieback disease on mango seedlings. The actinobacteria tested alone (BCA treatment) did not cause any harmful effects on mango fruits (Figure 3). This clearly showed that the antagonists and/or their metabolites(s) are able to inhibit the pathogen preventing it from producing lesions on the mango fruit surface.

### *In vitro* evaluation of antagonistic properties of the BCA candidates

The filter-sterilized crude culture filtrate of either BCA1 or BCA2 introduced into the wells using the cup plate technique, caused significant (*P* < 0.05) retardation of the growth of *L. theobromae*, when compared to the antifungal metabolite non-producing BCA3 or control (Table 3; Figure S1). Notably, the effect of diffused antifungal metabolites by BCA1 was significantly (*P* < 0.05) higher than those produced by BCA2 (Table 3).

**Table 3:** *In vitro* antagonistic activities of the three BCA candidates against *Lasiodiplodia theobromae*.

**Activities**	**BCA1**	**BCA2**	**BCA3**
Production of diffusible metabolites using the cup plate technique (diameter of zone of inhibition measured in mm)	67.97 ± 0.91 *a*	53.82 ± 0.93 *b*	0.00 ± 0.00 *c*
Production of diffusible metabolites using the dialysis membrane technique from the fish meal extract agar plates^a^	+	+	−
Production of chitinase using the dialysis membrane technique from the colloidal chitin agar plates^a^	+	−	+
Production of volatile compounds^a^	+	−	−
Production of hydrogen cyanide^b^	−	−	−
Production of siderophores^b^	+	−	−
Chitinase from colloidal chitin (U ml^−1^)^c^	5.56 ± 0.17 *a*	0.00 ± 0.00 *b*	7.75 ± 0.22 *c*
Chitinase from *L. theobromae* cell wall (U ml^−1^)^c^	3.73 ± 0.14 *a*	0.00 ± 0.00 *b*	5.60 ± 0.21 *c*
ß-1,3-glucanase from laminarin (U ml^−1^)^d^	3.57 ± 0.16 *a*	0.00 ± 0.00 *b*	5.54 ± 0.18 *c*
ß-1,3-glucanase from *L. theobromae* cell wall (U ml^−1^)^d^	2.79 ± 0.20 *a*	0.00 ± 0.00 *b*	3.76 ± 0.21 *c*

a *+, fungicidal effect; −, no fungicidal effect*.

b *+, produced; −, not produced*.

c *A unit of chitinase was expressed as the amount of the enzyme that released 1 μmol of N-acetyl-D-glucosamine mg^−1^ protein h^−1^*.

d *A unit of ß-1,3-glucanase was expressed as the amount of the enzyme that released 1 μmol of glucose mg^−1^ protein h^−1^*.

*Values are means of three replicates ± SE. Values with the same letter within a row are not significantly (P > 0.05) different according to Duncan's multiple range test*.

*BCA1, isolate #12; Streptomyces samsunensis UAE1; BCA2, isolate #29; S. cavourensis UAE1; BCA3, isolate #44; Micromonospora tulbaghiae UAE1*.

The growth of the pathogen was clearly inhibited by the diffused metabolites of BCA1 and BCA2 only after removing the dialysis membranes from the fish meal extract agar, compared to the control or BCA3 (Table 3; Figure S1). In addition, the pathogen failed to grow from the plugs transferred from the treatment plates to fresh PDA in the absence of diffused metabolites, confirming that the metabolites of BCA1 and BCA2 were clearly fungicidal to *L. theobromae*.

The diffused metabolites of BCA1 and BCA3 from the colloidal chitin agar plates, inhibited the growth of *L. theobromae* inoculum; in contrast to isolate BCA2 or control, after removing the dialysis membranes (Table 3; Figure S1). The pathogen did not recover from the plugs when transferred from treated plates to fresh PDA. This indicated that BCA1 and BCA3 showed fungicidal activities to *L. theobromae*.

To determine whether the BCA produced volatile antifungal compounds, the three BCA candidates were grown on fish meal extract agar. BCA2 and BCA3 failed to produce any volatile antifungal compounds, capable to inhibit the growth of the pathogen (Table 3; Figure S2). However, BCA1 produced volatile antifungal compounds and caused complete suppression of the pathogen growth. None of the three BCA candidates produced hydrogen cyanide; while only BCA1 produced siderophores (Table 3; Figure S2).

Chitinase production by BCA1 and BCA3 was significantly (*P* < 0.05) higher in the media amended with colloidal chitin or on media amended with the pathogen cell walls (Table 3). Both BCA1 and BCA3 also produced ß-1,3-glucanase when grown on media amended with laminarin or *L. theobromae* cell walls. The production of ß-1,3-glucanase was found to be significantly (*P* < 0.05) higher on laminarin-amended medium. On the other hand, there were no detectable levels of chitinase or ß-1,3-glucanase by BCA2 when it was grown in media containing either colloidal chitin or *L. theobromae* cell walls, or in media containing laminarin or *L. theobromae* cell walls, respectively (Table 3). The production of chitinase and ß-1,3-glucanase by BCA3 were, however, significantly (*P* < 0.05) higher than those produced by BCA1.

### Effect of crude culture filtrates of the BCA candidates on *L. theobromae*

We showed that the filter-sterilized crude culture filtrates of either BCA1 or BCA2 from fish meal extract broth were effective in inhibiting growth of *L. theobromae* (Table 4). On PDA plates, the increasing levels of the BCA1 and BCA2, but not BCA3, crude culture filtrates significantly (*P* < 0.05) inhibited the colony and mycelial growth of *L. theobromae* (Table 4; Figure S3). Mycelial growth was totally inhibited when crude culture filtrates were incorporated into PDA at 75% or above. In PDB, the assay of the mycelial growth inhibition of the pathogen by BCA1 and BCA2 was similar to that in PDA plates. Crude culture filtrates of BCA1 and BCA2 from fish meal extract broth significantly decreased the mycelial dry weight of the pathogen when proportionally added into PDB (Table 4). When compared with the control, the crude culture filtrates of BCA1 and BCA3 from colloidal chitin broth increasingly inhibited colony growth on PDA plates and mycelial dry weight of *L. theobromae* on PDB, with the increasing levels of crude culture filtrates after 5 days of incubation at 28°C (Table 4).

**Table 4:** Inhibition of mycelial growth, spore germination and germ tube elongation of *Lasiodiplodia theobromae* by the crude culture filtrate of the three BCA candidates either obtained from fish meal extract broth or colloidal chitin broth.

**BCA**	**Culture filtrate (%)**	**Colony diameter (mm)**	**Mycelial dry weight (g)**	**Conidia germination (%)**	**Germ tube length (μm)**
**(A) FISH MEAL EXTRACT BROTH**					
BCA1	0	99.60 ± 0.22**a**	81.05 ± 2.76**a**	90.06 ± 1.77**a**	59.86 ± 0.74**a**
	10	37.76 ± 1.39**b**	28.40 ± 2.86**b**	34.25 ± 1.74**b**	47.24 ± 0.92**b**
	25	18.87 ± 0.95**c**	9.86 ± 1.08**c**	21.31 ± 1.34**c**	29.35 ± 0.94**c**
	50	9.72 ± 0.84**d**	3.88 ± 0.76**d**	8.96 ± 0.36**d**	18.59 ± 1.58**d**
	75	0.00 ± 0.00**e**	0.40 ± 0.10**d**	3.19 ± 0.32**d**	11.81 ± 0.84**e**
	100	0.00 ± 0.00**e**	0.00 ± 0.00**d**	0.54 ± 0.09**d**	3.50 ± 0.61**f**
BCA2	0	99.51 ± 0.12**a**	78.60 ± 3.60**a**	89.17 ± 1.91**a**	53.26 ± 1.53**a**
	10	39.72 ± 1.83**b**	35.87 ± 2.08**b**	40.36 ± 3.03**b**	36.09 ± 1.91**b**
	25	21.57 ± 0.89**c**	15.92 ± 2.35**c**	26.12 ± 1.41**c**	28.17 ± 1.79**c**
	50	11.33 ± 1.21**d**	7.82 ± 1.15**d**	16.19 ± 0.91**d**	16.36 ± 0.01**d**
	75	0.00 ± 0.00**e**	3.15 ± 0.59**de**	6.68 ± 0.37**e**	6.32 ± 1.27**e**
	100	0.00 ± 0.00**e**	0.15 ± 0.08**e**	0.97 ± 0.02**f**	0.42 ± 0.14**f**
**(B) COLLOIDAL CHITIN BROTH**					
BCA1	0	98.70 ± 0.68**a**	80.39 ± 2.97**a**	93.10 ± 2.13**a**	60.69 ± 1.77**a**
	10	48.13 ± 2.05**b**	40.49 ± 1.66**b**	49.49 ± 2.86**b**	36.58 ± 1.85**b**
	25	26.94 ± 1.14**c**	22.80 ± 1.82**c**	28.55 ± 0.90**c**	25.11 ± 1.28**c**
	50	16.25 ± 1.23**d**	13.58 ± 1.03**d**	18.87 ± 0.93**d**	13.21 ± 1.29**d**
	75	0.00 ± 0.00**e**	4.24 ± 0.92**e**	8.48 ± 0.75**e**	3.41 ± 0.51**e**
	100	0.00 ± 0.00**e**	0.48 ± 0.16**e**	2.19 ± 0.42**f**	0.00 ± 0.00**e**
BCA3	0	99.84 ± 0.12**a**	83.27 ± 2.64**a**	93.32 ± 1.47**a**	66.34 ± 1.81**a**
	10	29.27 ± 1.45**b**	30.84 ± 2.79**b**	28.04 ± 0.86**b**	42.55 ± 1.49**b**
	25	16.79 ± 1.05**c**	13.12 ± 1.34**c**	18.42 ± 1.03**c**	17.44 ± 1.30**c**
	50	6.85 ± 1.00**d**	4.96 ± 0.95**d**	10.03 ± 0.81**d**	6.07 ± 0.83**d**
	75	0.00 ± 0.00**e**	0.00 ± 0.00**e**	2.49 ± 0.45**e**	0.00 ± 0.00**e**
	100	0.00 ± 0.00**e**	0.00 ± 0.00**e**	0.00 ± 0.00**e**	0.00 ± 0.00**e**

*Values are means of three replicates ± SE. Values with the same letter within a column for each BCA are not significantly (P > 0.05) different, according to Duncan's multiple range test*.

*BCA1, isolate #12; Streptomyces samsunensis UAE1; BCA2, isolate #29; S. cavourensis UAE1; BCA3, isolate #44; Micromonospora tulbaghiae UAE1*.

Similarly, a significant (*P* < 0.05) reduction in the germination of the thick walled, mature conidia and the average length of germ tubes produced by *L. theobromae* was found when the pathogen was exposed to the crude culture filtrate of BCA1 and BCA2 (in fish meal extract broth), and BCA1 and BCA3 (in colloidal chitin broth) after 24 h of incubation (Table 4). This indicates that the crude culture filtrates of BCA1, BCA2, and BCA3 inhibited not only mycelial growth, but also spore germination and germ tube elongation of *L. theobromae*.

**Figure 4:**
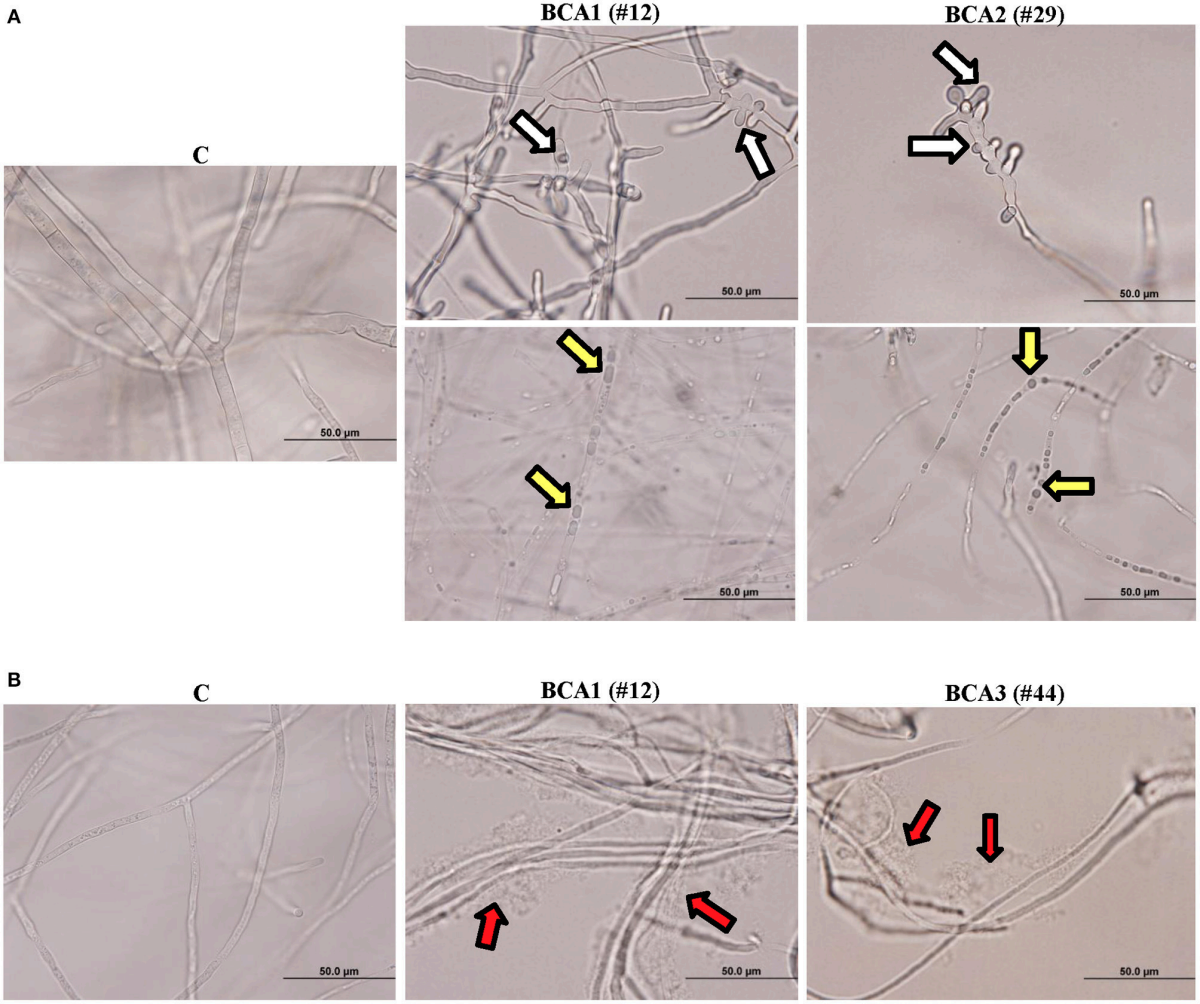
Effect of the BCA candidates on hyphae and cytoplasm of *Lasiodiplodia theobromae*. Abnormalities observed in hyphal morphology and cytoplasmic contents of *L. theobromae*, following exposure to **(A)** filter-sterilized crude culture filtrate of BCA1 (isolate #12; *Streptomyces samsunensis* UAE1) and BCA2 (isolate #29; *S. cavourensis* UAE1) on fish meal extract broth, or **(B)** BCA1 and BCA3 (isolate #44; *Micromonospora tulbaghiae* UAE1) on colloidal chitin broth compared to control. White arrows point to hyphal septum malformation and branch deformation; while yellow and red arrows point to cytoplasmic coagulation and lysis of cytoplasm, respectively.

We also observed hyphal abnormalities such as hyphal swelling (ballooning), cytoplasmic coagulation and hyphal lysis in *L. theobromae* treated with the crude culture filtrate of BCA1 obtained from fish meal extract broth and colloidal chitin broth, respectively (Figure 4). There were hyphal abnormalities, including hyphal swelling and cytoplasmic coagulation without hyphal lysis in *L. theobromae* exposed to the crude culture filtrate of BCA2 from fish meal extract broth (Figure 4A). The pathogen treated with the crude culture filtrate of BCA3 obtained from colloidal chitin broth showed only hyphal lysis (Figure 4B). Mycelial mats in control flasks remained healthy and unaffected (Figure 4).

### Identification of the promising BCA candidates to the species level

**Figure 5:**
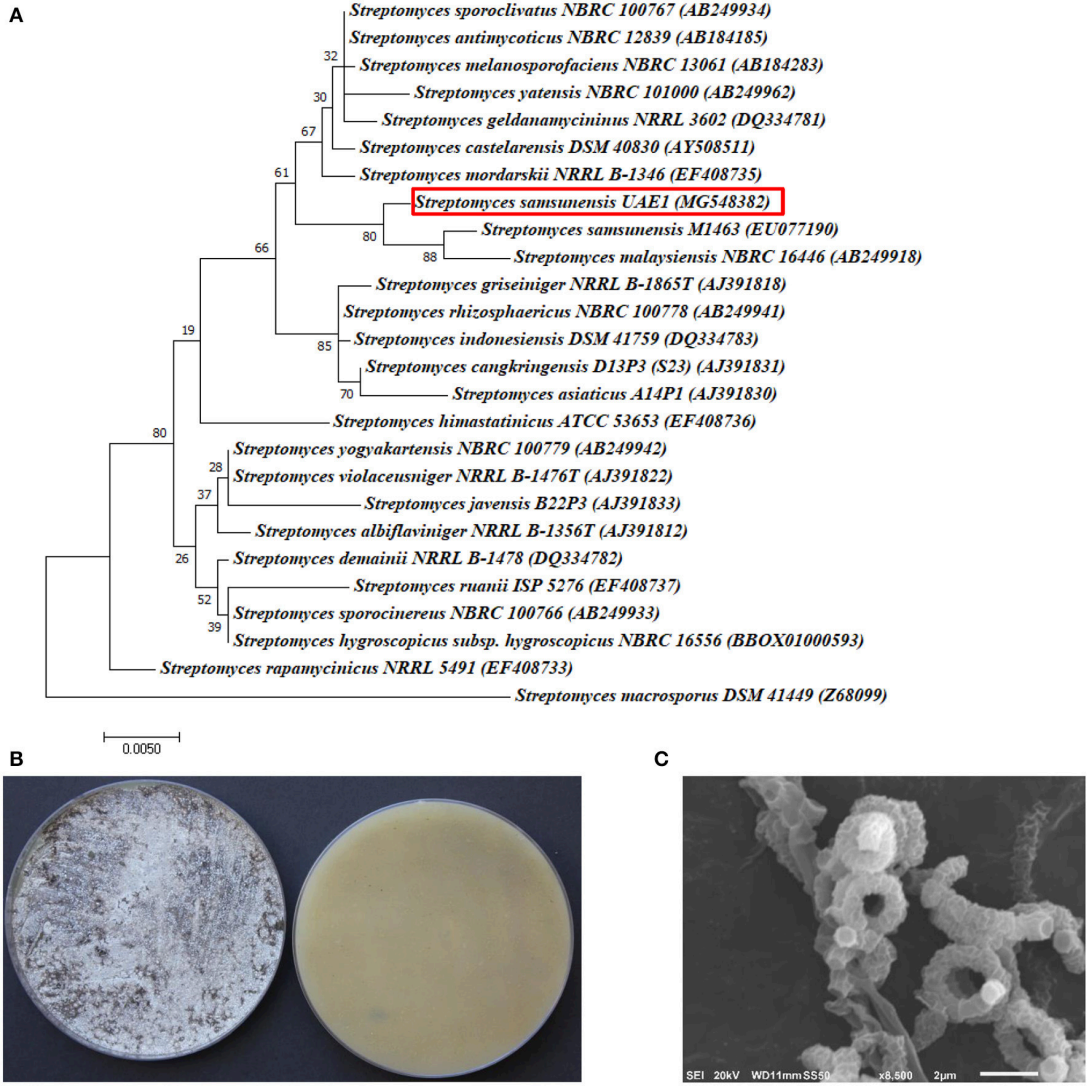
Taxonomic determination of *Streptomyces samsunensis* UAE1, based on phylogenetic, cultural and morphological characteristics. **(A)** The tree showing the phylogenetic relationships between *S. samsunensis* UAE1 (*MG548382*; 1,475 bp) and other members of *Streptomyces* spp. on the basis of 16*S* rRNA sequences. **(B)** Grayish black aerial mycelia (left) and grayish yellow substrate mycelia (right) growing on ISP medium 3 supplemented with yeast extract, **(C)** scanning electron micrograph (8,500X) showing the spiral chains of rugose ornamented spores of *S. samsunensis* UAE1 (BCA1; isolate #12). In **(A)** numbers at nodes indicate percentage levels of bootstrap support based on a maximum likelihood analysis of 1,000 resampled datasets. Bar, 0.005 substitutions per site. *S. macrosporus DSM 41449* (Z69099) was used as an outgroup. GenBank accession numbers are given in parentheses.

**Figure 6:**
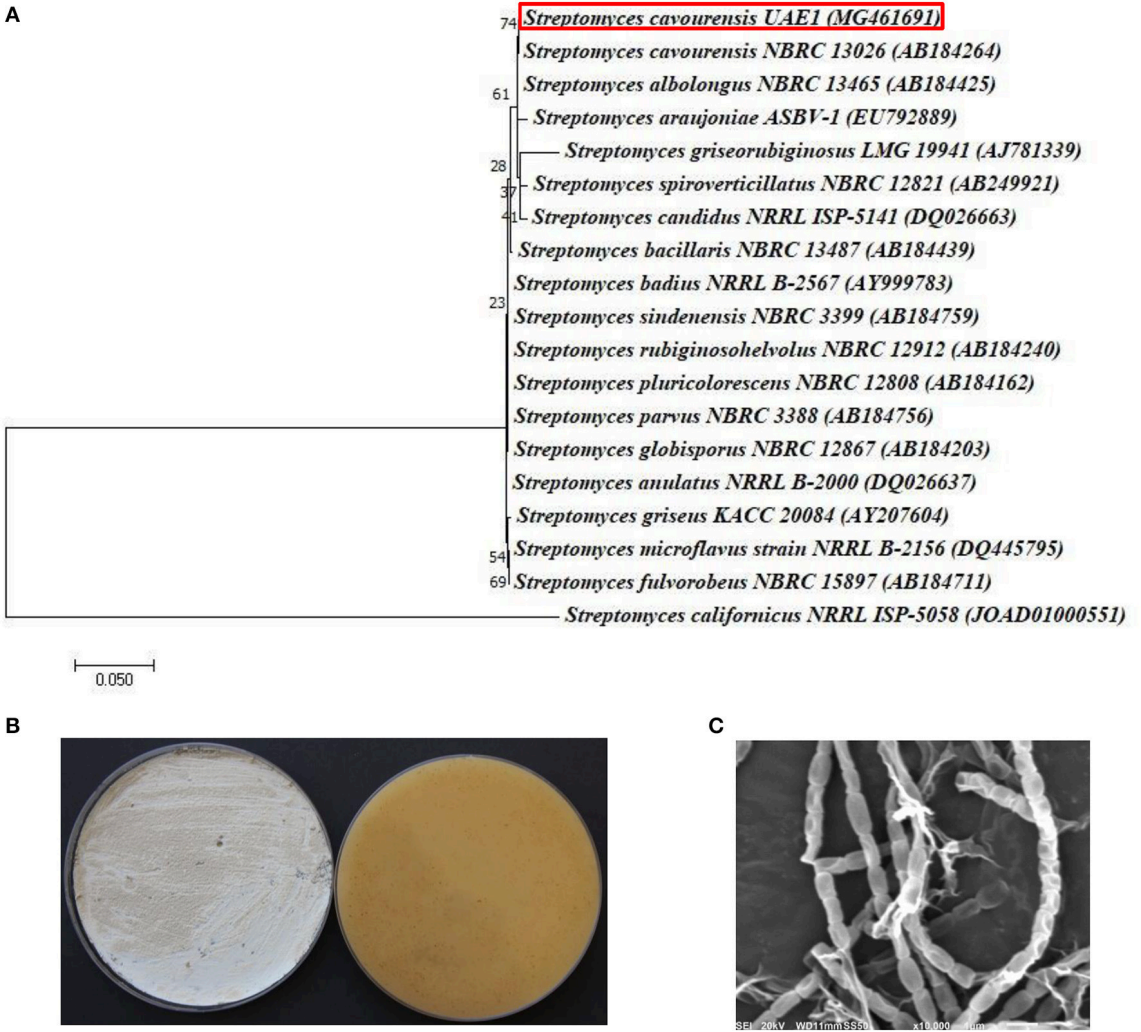
Identification of *Streptomyces cavourensis* UAE1, based on phylogenetic, cultural and morphological characteristics. **(A)** The tree displaying the phylogenetic relationships between *S. cavourensis* UAE1 (*MG461691*; 1,484 bp) and other members of *Streptomyces* spp. on the basis of 16*S* rRNA sequences. **(B)** Yellow aerial mycelia (left) and yellowish-brown substrate mycelia (right) growing on ISP medium 3 supplemented with yeast extract, and **(C)** scanning electron micrograph (10,000X) of the straight to flexuous (Rectiflexibiles) chains and smooth-surfaced spores of the strain of *S. cavourensis* UAE1 (BCA2; isolate #29). In **(A)** numbers at nodes indicate percentage levels of bootstrap support based on a maximum likelihood analysis of 1000 resampled datasets. Bar, 0.05 substitutions per site. *S. californicus* NRRL ISP-5058 (JOAD0100051) was used as an outgroup. GenBank accession numbers are given in parentheses.

**Figure 7:**
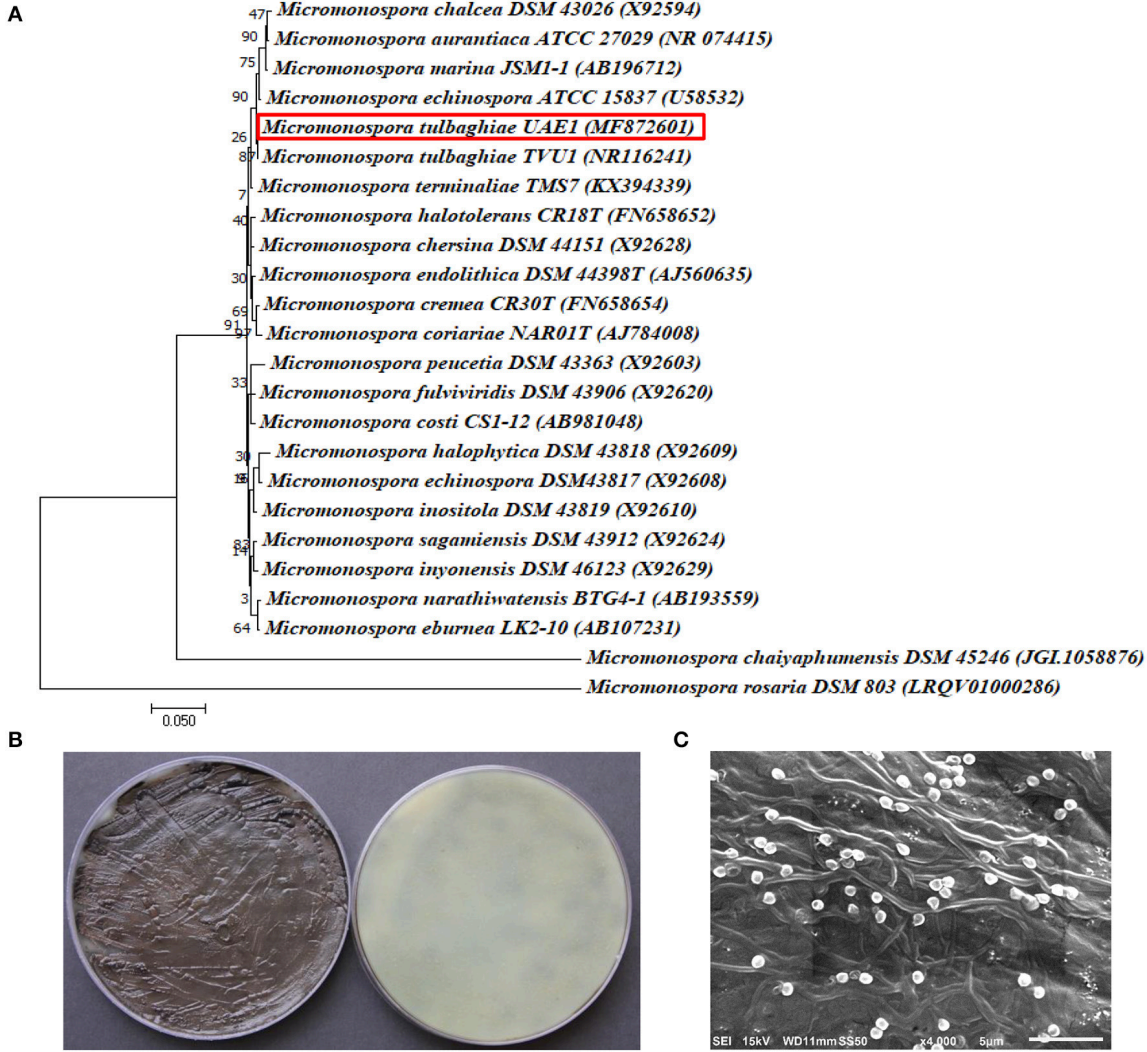
Identification of *Micromonospora tulbaghiae* UAE1 using phylogenetic, cultural and morphological characteristics. **(A)** The tree shows the phylogenetic relationships among *M. tulbaghiae* UAE1 (*MF872601*; 1,486 bp) and other members of *Micromonospora* spp. on the basis of 16*S* rRNA sequences. **(B)** Brownish-black charcoal-like substrate mycelia without the formation of aerial mycelium (left) and the brownish-black color of the substrate mycelia (right) growing on ISP medium 3 supplemented with yeast extract, and **(C)** scanning electron micrograph (4,000X) of the single oval to spherical smooth-surfaced spores of the strain of *M. tulbaghiae* UAE1 (BCA3; isolate #44). In **(A)** numbers at nodes indicate percentage levels of bootstrap support based on a maximum likelihood analysis of 1,000 resampled datasets. Bar, 0.05 substitutions per site. *M. rosaria* DSM 803 (LRQV01000286) was used as an outgroup. GenBank and Joint Genome Institute (JGI) accession numbers are given in parentheses.

The promising antagonists BCA1, BCA2, and BCA3 were identified by determining the nucleotide sequence of their 16*S* rRNA gene. The 16*S* rRNA gene sequences of BCA1 (*Streptomyces samsunensis*; GenBank accession number MG548382), BCA2 (*S. cavourensis*; MG548383) and BCA3 (*Micromonospora tulbaghiae*; MG548384) were compared with that of other actinobacteria. Comparisons of the 16*S* rRNA gene of BCA1, BCA2 and BCA3 with sequences in the GenBank database showed that these BCA candidates were streptomycete spp. for isolates #12 (BCA1) and #29 (BCA2) and a non-streptomycete sp. for isolate #44 (BCA3). BCA1 showed above 99% similarity to *S. samsunensis* (EU077190) and *S. malaysiensis* (AB249918) (Figure 5A), although, the remaining isolates of *Streptomyces* spp. showed less than 98.8% similarities. The phylogenetic analysis of BCA2 showed 100% similarity to both *S. cavourensis* (AB184264) and *S. albolongus* (AB184425) (Figure 6A); while the rest showed < 99.6% similarity with the strain of interest. This may suggest that BCA1 may possibly be either *S. samsunensis* or *S. malaysiensis*; while BCA2 could be either *S. cavourensis* or *S. albolongus*; thus it was necessary to obtain a more reliable identification of these isolates. Based on the 16*S* rRNA gene comparisons, the third identified BCA3 was considered as *Micromonospora tulbaghiae* (Kirby and Meyers, 2010) Strain UAE1, due to the high similarity (100%) with *M. tulbaghiae* (DSM 45142). The rest of the *Micromonospora* spp. showed 99.4% or less similarity than that with this specific strain (Figure 7A).

To confirm the identity of BCA1, the pure cultures produced grayish black aerial mycelia with grayish yellow substrate mycelial growth on ISP medium 3 for 14 days (Figure 5B). Using SEM, the configuration of the spore chains showed spiral chains of rugose ornamented spores (Figure 5C). Together, the genotypic, morphological, cultural and phenotypic data showed that BCA1 can be recognized as *Streptomyces samsunensis* (Sazak et al., 2011) Strain UAE1 (Table S1).

On the other hand, typical yellow aerial mycelia and yellowish-brown substrate mycelia were observed when BCA2 was cultivated (Figure 6B). BCA2 showed straight to flexuous (Rectiflexibiles) chains and smooth-surfaced spores (Figure 6C). Our data demonstrated that BCA2 (isolate #29) can be recognized as *Streptomyces cavourensis* (Giolitti, 1958) (in Waksman, 1961) Strain UAE1 (Table S2). Pure cultures of BCA3 (*M. tulbaghiae*) on ISP medium 3 produced typical brownish-black charcoal-like substrate mycelia without the formation of aerial mycelia (Figure 7B); with the formation of single oval to spherical smooth-surfaced spores (Figure 7C).

### Effect of the promising BCA candidates on *L. theobromae* in the greenhouse

The responses of the pathogen to these selected antagonists *in vitro* clearly indicated the three BCA candidates to be effective against mango dieback caused by *L. theobromae*. With an aim of evaluating and comparing the outcome of the application of the BCAs in suppressing *L. theobromae* using different antifungal activities, an *in vivo* experiment involving greenhouse grown plants was conducted. For this purpose, “preventive” treatments with the three BCA candidates 1 week before inoculation with *L. theobromae* were applied to determine their impact on mango dieback.

**Figure 8:**
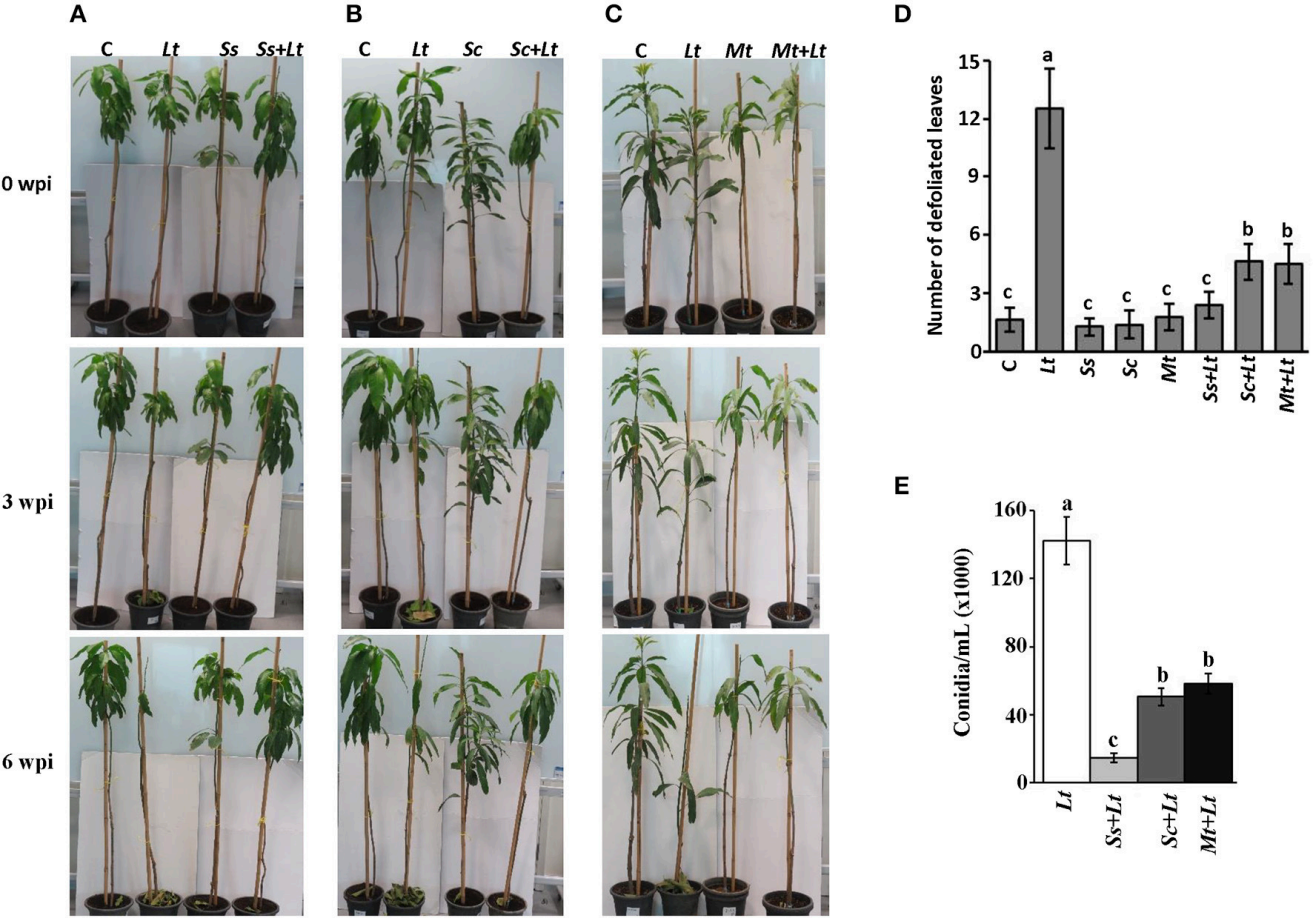
Antagonistic effect of BCA candidates against mango dieback disease under greenhouse conditions. Effect of preventive biocontrol treatment of **(A)** *Streptomyces samsunensis* UAE1 (BCA1; isolate #12); **(B)** *S. cavourensis* UAE1 (BCA2; isolate #29); and **(C)** *Micromonospora tulbaghiae* UAE1 (BCA3; isolate #44). The number of **(D)** defoliated leaves; and **(E)** conidia after recovery of the pathogen from affected mango stem tissues (cv. Badami) at 6 and 9 wpi with *L. theobromae*, respectively. In **(D,E)** values with different letters are significantly different from each other at *P* < 0.05. C, non-inoculated control seedlings, *Lt*, seedlings inoculated with *L. theobromae* only; *Ss, Sc* or *Mt*, seedlings inoculated with only *S. samsunensis* UAE1, *S. cavourensis* UAE1 or *M. tulbaghiae* UAE1, respectively; *Ss*+*Lt, Sc*+*Lt* or *Mt*+*Lt*, seedlings inoculated with the individual BCA, *S. samsunensis* UAE1*, S. cavourensis* UAE1 or *M. tulbaghiae* UAE1, respectively, 1 week prior to *L. theobromae* inoculation; wpi, weeks post inoculation.

Initially, a pathogenicity test was carried out to determine the effect of inoculation with *L. theobromae* on mango seedlings. Symptoms typical of the mango disease after 3 wpi with *L. theobromae* (*Lt*) were observed (Figures 8A–C). The disease progressed with time, initially with leaves of infected seedlings showing distinct defoliation at 6 wpi. No disease symptoms were evident in any of the plants inoculated with BCA candidate alone (*Ss, Sc*, or *Mt*) or on non-inoculated seedlings (C) (Figures 8A–C). Secondly, we individually applied the BCA candidates *S. samsunensis, S. cavourensis* or *M. tulbaghiae* on seedlings 1 week before inoculation with *L. theobromae*, designated as *Ss*+*Lt, Sc*+*Lt*, or *Mt*+*Lt*, respectively. Plants inoculated with the BCA candidates prior to inoculation with *L. theobromae* (*Ss*+*Lt, Sc*+*Lt*, or *Mt*+*Lt*) recovered when compared with seedlings inoculated with *L. theobromae* only (*Lt*) at all time points of assessments. These plants appeared to be healthy and were comparable to those that were inoculated with any of the corresponding BCA candidate alone (*Ss, Sc*, or *Mt*) or without infection with *L. theobromae* (C) (Figures 8A–C; Figure S4). This suggests that these BCA candidates effectively inhibit *L. theobromae* growth *in vivo*.

We found significant differences between treatments when the DSI was assessed. Plants inoculated with *L. theobromae* (*Lt*) showed disease progression until 9 wpi (Table S3). Seedlings not inoculated with *L. theobromae* (control; C) showed no disease symptoms at any of the assessment time-points. There was a dramatic decrease in DSI in all *L. theobromae*-infected seedlings that were previously treated with any of the BCA candidates at 3 and 9 wpi, when compared with plants inoculated with the pathogen alone. In comparison with the *L. theobromae*-infected seedlings, the DSI of the preventive applications of individual BCA candidate (*Ss*+*Lt, Sc*+*Lt*, and *Mt*+*Lt*) dropped from 2.83 to 0.17 at 3 wpi and from 4.33 to 0.17 at 9 wpi; providing 94.0 and 96.1% reduction in disease development, respectively. The three preventive treatments with the BCA candidates at 3 and 9 wpi did not show significant (*P* > 0.05) difference among the DSI measurements, and the BCA-treated plants without the pathogen. This suggests that the preventive treatment with BCA candidates a week before inoculation with *L. theobromae* effectively suppresses the pathogen invasion.

It is evident that the DSI values in the case of BCA applications were significantly lower than those with *L. theobromae*; however, the differences of the DSI were indistinguishable among these BCA treatments. For that reason, we compared the responses of the biological control treatments to determine their effects on leaf defoliation and the production of conidia on the host plant. The number of falling leaves in seedlings inoculated with *L. theobromae* dramatically increased compared to any BCA-inoculated or non-inoculated seedlings at 6 wpi (Figure 8D). Mango seedlings treated with BCA before *L. theobormae* infection significantly reduced the number of defoliated leaves; thus seedlings of *Ss*+*Lt* showed the lowest number of falling leaves among the other two treatments and were comparable to its corresponding *Ss* treatment. In addition, conidia counts of *L. theobromae* at the leaf base of treated mango seedlings were made. The BCA treatment (*Ss*+*Lt*) caused a greater reduction in the number of conidia, followed by the other BCA-treated plants, *Sc*+*Lt* and *Mt*+*Lt* at the end of the greenhouse experiment (Figure 8E). At least 4-fold reduction in total conidia numbers of *L. theobromae* in *S. samsunensis*-treated plants was measured when compared with other BCA treatments (Figure 8E). In general, the pathogen appeared not to be sufficiently aggressive to support disease progression in the presence of *S. cavourensis* or *M. tulbaghiae*, while a strong inhibitory effect on the pathogen was observed in the case of the *S. samsunensis* treatment. Together, this suggests that *S. samsunensis*, which exhibited multiple modes of action, successfully controlled mango dieback disease.

## Discussion

Nowadays, seeking an alternative strategy to the use of chemicals by native microorganisms as a source material for plant growth promotion and disease amelioration in agriculture has become a priority (Saeed et al., 2017b; Syed Ab Rahman et al., 2018). Actinobacteria could be potential targets as BCA candidates since they have many properties that control diseases, increase nutrient supply and enhance growth of plants (Doumbou et al., 2001; Barka et al., 2016). Ultimately, this approach can be employed as a main component in IPM, and may be combined with others to prevent losses and damages caused by plant diseases (Saeed et al., 2017b; Syed Ab Rahman et al., 2018). These outcomes led us to explore local UAE soils for the actinobacterial communities and screen their potential under *in vitro* and *in vivo* greenhouse conditions for their effectiveness in the control of the mango dieback disease caused by *L. theobromae*. We hypothesized that exposure to microbial metabolites produced by the soil-inhabiting actinobacteria can be exploited for the management of this devastating mango disease.

In this study, we aimed at identifying SA and NSA isolates that are capable of restricting invasions of *L. theobromae* on mango plantations. A total of 53 actinobacterial strains were obtained from the rhizosphere of healthy mango plants. Due to their dominance as a biologically active component of the soil microflora, many researchers have successfully isolated SA from soil environments (Goodfellow and Williams, 1983; Palaniyandi et al., 2013). The NSA, however, are rarely isolated actinobacteria whose isolation frequency using commonly used techniques is usually lower than the numbers of SA (Jose and Jebakumar, 2013). For that reason, the isolation of the uncommon actinobacteria was achieved by the application of *Streptomyces* polyvalent phages that selectively permitted the appearance of the genera of NSA on isolation plates, a technique not commonly used in screening for BCA candidates. Some studies have recommended the use of *Streptomyces* phages to omit “weedy” *Streptomyces* colonies (Kurtböke et al., 1992; Kurtböke, 2012). In the current study, the isolated NSA group comprised of *Actinoplanes, Actinomadura, Microbispora, Micromonospora, Nocardia, Rhodococcus*, and *Streptosporangium* spp. (Table 1).

The development of new BCA and/or biocontrol products for use against plant diseases requires screening of large numbers of antagonistic candidates (Köhl et al., 2011). In our effort to conduct an appropriate performance evaluation of BCA candidate(s) against *L. theobromae*, a series of screening steps were performed. A first round of *in vitro* screening on agar plates allowed rapid and clear discriminating results. Secondly, a selection of antagonists to *L. theobromae*, using the novel *in vivo* mango fruit bioassay was also evaluated. Thirdly, selected candidates were identified to the genus and species levels. Finally, an assessment of the feasibility of the selected BCA candidates in controlling dieback disease on mango seedlings under greenhouse conditions was carried out. Accordingly, 11 were regarded as highly diffusible antifungal metabolite-producing isolates on fish meal extract agar plates. In addition, an agar medium incorporating *L. theobromae* mycelial fragments helped to select 12 antagonists which had the required enzymes to destroy the components of *L. theobromae* cell wall. Mycelial fragment agar has been previously used to isolate glucanolytic BCA candidates against *Phytophthora fragariae* (Valois et al., 1996) and *Pythium aphanidermatum* (El-Tarabily, 2006). Many promising antagonistic isolates obtained in the current investigation that showed clearing zones on *L. theobromae* mycelial fragment agar and on colloidal chitin agar, may have secreted ß-1,3-glucanases and chitinase which hydrolyzed glucans and chitin present in the pathogen cell wall and helped to lyse *L. theobromae* hyphae. This study clearly indicates that SA and NSA can serve as potential BCAs against *L. theobromae*.

Because testing of isolates under greenhouse and field conditions for efficacy requires additional labor and time, the antagonistic potential of candidates were further assessed in a second screening round of laboratory bioassays. We argue that the mango fruit antagonism bioassay would provide a “clear-cut” prediction of what may occur in the field. Such laboratory *in vivo* assays using plant material are of value since they provide a rapid screening of large numbers of antagonistic candidates in the presence of the pathogen. Thus, the results must be confirmed by greenhouse/field trials. Previous studies have used similar “bioassay” approach as a practical step in the selection of BCAs for major pathogens and pests on crops. In assays carried out *in vitro* as well as on the carrot roots or mango fruits, the BCAs used were found to be capable of almost complete inhibition of *P. coloratum* or *L. theobromae*, respectively (El-Tarabily et al., 1997; Seethapathy et al., 2016). Seethapathy et al. (2016) have demonstrated that dual culture technique of bacterial anatogonists using *Pseudomonas fluorescens* (Pf1) and *Bacillus subtilis* (EPCO16) reduced the pathogen population *in vitro*; and further strengthened the cell-wall structures of mango fruits against *L. theobromae* infection. This is consistent with the present study that many isolates killed the pathogen *in vitro*; whereas certain isolates completely arrested development of lesions, with others reducing only the lesion size or having no effect on the disease in the mango fruit bioassay (Table 2). According to El-Tarabily et al. (1997), the failure of isolates to reduce lesion diameter in the mango fruit bioassay indicated that their ability to produce antifungal metabolites in agar did not necessarily address that this performance would be reproducible on plant material. Our data indicate the importance of the *in vitro* as well as the mango fruit bioassay for the selection of potential antagonists prior to their screening on plants in the greenhouse.

The efficacious isolates from the *in vitro* and *in vivo* assays were further identified. The three candidates represent 5.7% of the total isolated actinobacteria from the soil of healthy mango rhizosphere. In this study, the two identified isolates (#12 and #29) belonging to *Streptomyces* spp. confirm the findings from previous reports that SA are predominant among actinobacteria appearing in isolation plates and more often produce useful antibiotics and active secondary metabolites (Thenmozhi and Krishnan, 2011; Barka et al., 2016). Isolates #12 (BCA1) and #29 (BCA2) were identified as *S. samsunensis* and *S. cavourensis*, respectively, while BCA3 (isolate #44) was considered as *M. tulbaghiae*. The mechanisms involved in disease reduction or prevention of lesion development appeared to be antibiosis (diffusible antifungal metabolites and volatile compounds) for *S. samsunensis* and *S. cavourensis*, and the production of CWDEs such as chitinase and ß-1,3-glucanases for *S. samsunensis* and *M. tulbaghiae*. Several actinobacteria have been demonstrated to inhibit the growth of soil-borne plant pathogens such as *Pythium ultimum, Rhizoctonia solani* (Yuan and Crawford, 1995), *P. coloratum* (El-Tarabily et al., 1997) and *Thielaviopsis punctulata* (Saeed et al., 2017b) *in vitro* via the production of inhibitory diffusible antifungal metabolites.

The cell walls of filamentous fungi consist largely of chitin and ß-glucans (Osherov and Yarden, 2010), it is probable that ß-1,3-glucanase and chitinases produced by the antagonistic actinobacteria in this study may be involved in the pathogen suppression. The exposure of phytopathogenic fungi to CWDEs can result in the lysis and degradation of the fungal cell walls (Doumbou et al., 2001; Whipps, 2001; Berini et al., 2018). Therefore, the production of chitinase and ß-1,3-glucanase was set, in this study, as a criteria for selection of potential BCA against *L. theobromae*. Chitinase-producing actinobacteria tested previously under *in vitro* conditions have included *Streptomyces viridicans* (Gupta et al., 1995), *S. viridodiasticus* (El-Tarabily et al., 2000), and *Streptomyces* spp. (Singh and Gaur, 2016). In addition, several chitinase-producing SA such as *Streptomyces* spp. (Singh et al., 1999), and NSA such as *Micromonospora carbonacea* (El-Tarabily et al., 2000) and *Actinoplanes missouriensis* (El-Tarabily, 2003) were used for the management of cucumber wilt caused by *Fusarium oxysporum* f.sp. *cucumerinum*, lettuce basal drop caused by *Sclerotinia minor*, and lupin root rot caused by *Plectosporium tabacinum*, respectively. Valois et al. (1996) have reported some ß-glucanase-producing actinobacterial isolates that hydrolyzed cell wall glucans, caused hyphal lysis and resulted in the suppression of root rot disease of raspberry caused by *Phytophthora fragariae*.

Beside its ability to produce diffusible and volatile inhibitory antifungal compounds and siderophores, *S. samsunensis* produced CWDEs. Better control of mango dieback disease by *S. samsunensis* in comparison to the other BCA candidates (*S. cavourensis* or *M. tulbaghiae*) may indicate that this response could be a result of synergetic effects of the multiple modes of action i.e., co-antagonism. Our results are in agreement with other reports that *Streptomyces spiralis* and *Actinoplanes campanulatus* which produced diffusible inhibitory antifungal metabolites and CWDEs were superior on *Micromonospora chalcea* which produced CWDEs only in controlling root rot and crown rot of cucumber caused by *P. aphanidermatum* (El-Tarabily et al., 2009).

Bailey and Falk (2011) have stated that less than 1% of candidate microorganisms isolated by routine isolations make successful antiobiotic products and secondary metabolites. Among these, *Streptomyces griseoviridis* K61 (Mycostop®), *S. lydicus* strain WYEC108 (Actinovate®, Actino-iron®, or Micro108® and *S. saraceticus* KH400 (YAN TEN) are commercial BCA products that reduce spore germination and inhibit hyphal growth of plant fungal pathogens (Minuto et al., 2006; Elliott et al., 2009; Palaniyandi et al., 2013). In the UAE, *Streptomyces globosus* UAE1 has recently been reported as the first effective BCA against black scorch disease in date palm plantations (Saeed et al., 2017b). On the other hand, few NSA have been recognized as BCA and/or PGP (El-Tarabily et al., 1997; El-Tarabily and Sivasithamparam, 2006). This suggests that the SA and NSA strains isolated in our study may also serve as producers of potentially useful antifungal products active against *L. theobromae*. Therefore, efforts to be among the first to manage mango dieback by the three antagonists were aimed in greenhouse trials.

One should take cautions when assuming a correlation between *in vitro* inhibition and greenhouse or field performance (Fravel, 2005; Parnell et al., 2016). In the present study, the actinobacteria were inoculated at the apices of the mango stems 1 week before inoculation with the pathogen. The gap in the incubation periods may favor the establishment of the introduced actinobacteria prior to the exposure to the pathogen, and/or to enable them to propagate on the mango stem or to activate the mechanism(s) of antagonism (Rothrock and Gottlieb, 1984). Prevention of infection using actinobacteria is a highly recommended management strategy in plant-pathogen interaction systems (Saeed et al., 2017b). It was clear that the application of any of the BCAs prior to pathogen invasion helped to establish the required biomass of the BCA; and thus delay the systemic invasion by *L. theobromae* within the host plant (Figure 8). The results obtained from the DSI indicated that *S. samsunensis, S. cavourensis*, or *M. tulbaghiae* have good potential as BCAs of the mango dieback caused by *L. theobromae*; yet it was difficult to choose the most effective BCA. With all the successful BCA applications, a significant reduction in disease symptoms regarding the number of falling leaves at 6 wpi and the conidia counts in affected tissues at 9 wpi in BCA1-treated seedlings was found. This suggests that *S. samsunensis* is the most efficient BCA among the tested strains, and may serve as a candidate biofungicide for the control of *L. theobromae*-affected mango orchids. To a lesser degree, *S. cavourensis* and *M. tulbaghiae* were also notably effective in reducing the pathogenicity of *L. theobromae* in the greenhouse.

Biological control can be used as an alternative method to agrochemicals if the BCA or its product survives temporarily adverse conditions and preferably improves plant performance (El-Tarabily and Sivasithamparam, 2006). The antagonistic *Streptomyces* and *Micromonospora* spp. identified in this study are safe, inexpensive, long lasting and well-suited to extreme harsh conditions, meeting the expectations previously reported in literature (Goodfellow and Williams, 1983; Ningthoujam et al., 2009). These indigenous strains of BCA are well-adapted to the local conditions of the UAE of dry soils and arid environments. In addition, actinobacteria are capable of producing spores resisting heat and drought stresses (Goodfellow and Williams, 1983), making them adequately suitable for being implemented as a prime component in IPM leading to sustainable agriculture in the future. In general, it is well-known that the CFU of actinobacteria remains high as soils dry out; while the relative incidence of bacteria is adversely affected as they lack tolerance to arid conditions (Alexander, 1977).

Together, the diffusible compounds and/or CWDEs produced by the BCA candidates were closely associated with the inhibition, suppression and destruction of *L. theobromae* within the plant host. The mechanisms identified are most likely to have significantly contributed to the relative success of these selected strains as BCAs. Other factors, such as the production of compounds capable of inducing host resistance (e.g., ISR) to the pathogen by the BCA (Martínez-Hidalgo et al., 2015) could also be attributed to the reduction of the mango dieback incidence detected. This form of resistance by the BCA candidates was not investigated in this research, but will surely be looked at in future studies. The present study provided the first record of SA and NSA as microbial antagonists to control a *Lasiodiplodia* disease. To our knowledge, the results demonstrate, for the first time, the isolation, identification and confirmation of the biocontrol potential of actinobacteria from soil native to the UAE, and likely to be naturally suited to be suppressive to *L. theobromae*. In order to make biocontrol more effective, future research focusing on development of novel formulations, broadening of host range targets, and increasing biomass production of BCAs in association with the use of biotechnology in improvement of BCA mechanisms will effectively develop schemes to widely manage the dieback disease using environmentally sustainable strategies.

## Author contributions

KE-T and SA designed the research and supervised the study. FK, ES, and KE-T performed *in vitro* and *in vivo* experiments. ES, KE-T, and SA performed *in vivo* greenhouse experiments. SA developed the phylogenetic analysis. KE-T and SA analyzed the data. FK and ES assisted with experiments and/or data evaluation. KE-T and SA wrote the manuscript. All authors critically revised the manuscript and approved the final version.

### Conflict of interest statement

The authors declare that the research was conducted in the absence of any commercial or financial relationships that could be construed as a potential conflict of interest.
