# Supplementary material for: Biological Control of Mango Dieback Disease Caused by Lasiodiplodia theobromae Using Streptomycete and Non-streptomycete Actinobacteria in the United Arab Emirates
Source: Front Microbiol. 2018 May 4;9:829. doi: 10.3389/fmicb.2018.00829 (PMC5945903; doi:10.3389/fmicb.2018.00829)
Supplement: Supplementary file 1 [file Data_Sheet_1.docx]

Supplementary Data

Biological Control of Mango Dieback Disease Caused by *Lasiodiplodia theobromae* Using Streptomycete and Non-streptomycete Actinobacteria in the United Arab Emirates

**Fatima H. Kamil,** **Esam Eldin Saeed, Khaled A. El-Tarabily*, and** **Synan F. AbuQamar***

*** Correspondence:**

Dr. Khaled El-Tarabily: [ktarabily@uaeu.ac.ae](mailto:ktarabily@uaeu.ac.ae)

Dr. Synan AbuQamar: [sabuqamar@uaeu.ac.ae](mailto:sabuqamar@uaeu.ac.ae)

# Supplementary Figures

**Figure S1.** Effect of the BCA candidates on mycelial growth of *Lasiodiplodia theobromae*. Cup plate method (A); and dialysis membrane overlay technique using (B) fish meal extract agar or (C) colloidal chitin agar plates. In (A & B), inhibition of *L. theobromae* mycelial growth was observed only by the diffusible antifungal metabolite-producing isolate *Streptomyces samsunensis* UAE1 (BCA1; isolate #12) and *S. cavourensis* UAE1 (BCA2; isolate #29) compared to the non-diffusible antifungal metabolite-producing isolate *Micromonospora tulbaghiae* UAE1 (BCA3; isolate #44). In (C), inhibition of *L. theobromae* mycelial growth were only by the chitinase-producing isolates #12 and #44 compared to the chitinase non-producing isolate #29. In (A), wells were inoculated with either filter-sterilized fish meal extract broth (C), or filter-sterilized crude culture filtrates of isolates #12, #29 or #44; while in (B & C), fish meal extract agar or colloidal chitin agar plates are either colonized by no BCA (C), or isolates #12, #29 or #44.

**Figure S2.** Effect of the BCA-producing volatile antifungal compounds on *Lasiodiplodia theobromae* and siderophore production. (A) Inhibition of *L. theobromae* mycelial growth by the volatile antifungal compound-producing isolate *Streptomyces samsunensis* UAE1 (BCA1; isolate #12) compared to the non-volatile antifungal compounds producing isolate *S. cavourensis* UAE1 (BCA2; isolate #29) and *Micromonospora tulbaghiae* UAE1 (BCA3; isolate #44). (B) Siderophore production by isolate #12 compared to the siderophore non-producing isolates #29 and #44 on chrome azurol S agar plates. In (A), from upper to lower panel, fish meal extract agar plates (right) are either colonized by no BCA (C), or isolates #12, #29 or #44. In (B), the yellow halo surrounding the colony indicates the excretion of siderophore.

**Figure S3.** Effect of filter-sterilized crude culture filtrates of BCA candidates on *Lasiodiplodia theobromae*. Gradual inhibition of *L. theobromae* colony growth on PDA plates containing different proportions (%) of crude culture filtrate obtained from fish meal extract broth only with the antifungal metabolite-producing isolates **(A)** *Streptomyces samsunensis* UAE1 (BCA1; isolate #12) and **(B)** *S. cavourensis* UAE1 (BCA2; isolate #29) compared to **(C)** the non-diffusible antifungal metabolite-producing isolate *Micromonospora tulbaghiae* UAE1 (BCA3; isolate # 44).

**Figure S4.** Antagonistic effect of BCA candidates against mango dieback disease caused by *Lasiodiplodia theobromae* in the greenhouse. Effect of preventive biocontrol treatment of (A) *Streptomyces samsunensis* UAE1 (BCA1; isolate #12); (B) *S. cavourensis* UAE1 (BCA2; isolate #29); (C) *Micromonospora tulbaghiae* UAE1 (BCA3; isolate #44) on mango seedlings at 9 wpi with *L. theobromae*. C, non-inoculated control seedlings, *Lt*, seedlings inoculated with *L. theobromae* only; *Ss*, *Sc* or *Mt,* seedlings inoculated with only *S. samsunensis*, *S. cavourensis* or *M. tulbaghiae*, respectively; *Ss*+*Lt*, *Sc*+*Lt* or *Mt*+*Lt*, seedlings inoculated with the individual BCA, *S. samsunensis, S. cavourensis* or *M. tulbaghiae*, respectively, one week prior to *L. theobromae* inoculation. wpi, weeks post inoculation.

**Table S1.** Comparison of morphological, cultural and phenotypic characteristics that distinguish BCA1 (isolate #12) from very closely related species *Streptomyces samsunensis* and *S. malaysiensis.*

**Table S2.** Comparison of morphological, cultural and phenotypic characteristics that distinguish BCA2 (isolate #29) from very closely related species *Streptomyces cavourensis* and *S. albolongus*.

**Table S3.** Disease severity index (DSI) of *Lasiodiplodia theobromae* inoculated-mango seedlings (cv. Badami) inoculated with each BCA candidate at 3 and 9 wpi (*n* = 6).

**
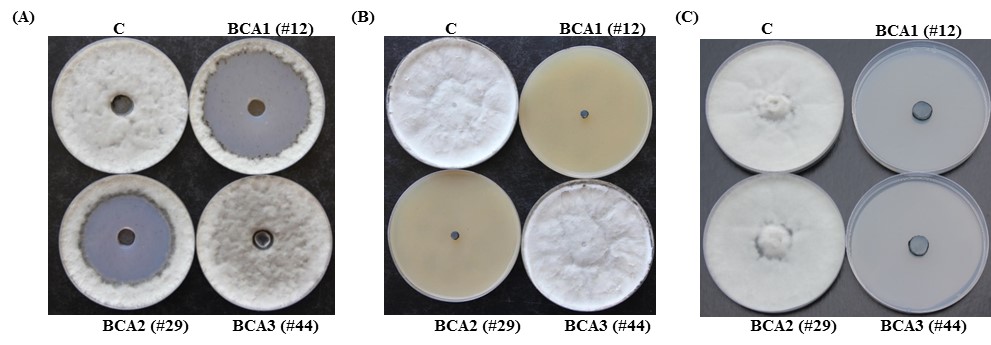
**

**Figure S1.** Effect of the BCA candidates on mycelial growth of *Lasiodiplodia theobromae*. Cup plate method (A); and dialysis membrane overlay technique using (B) fish meal extract agar or (C) colloidal chitin agar plates. In (A & B), inhibition of *L. theobromae* mycelial growth was observed only by the diffusible antifungal metabolite-producing isolate *Streptomyces samsunensis* UAE1 (BCA1; isolate #12) and *S. cavourensis* UAE1 (BCA2; isolate #29) compared to the non-diffusible antifungal metabolite-producing isolate *Micromonospora tulbaghiae* UAE1 (BCA3; isolate #44). In (C), inhibition of *L. theobromae* mycelial growth were only by the chitinase-producing isolates #12 and #44 compared to the chitinase non-producing isolate #29. In (A), wells were inoculated with either filter-sterilized fish meal extract broth (C), or filter-sterilized crude culture filtrates of isolates #12, #29 or #44; while in (B & C), fish meal extract agar or colloidal chitin agar plates are either colonized by no BCA (C), or isolates #12, #29 or #44.

**
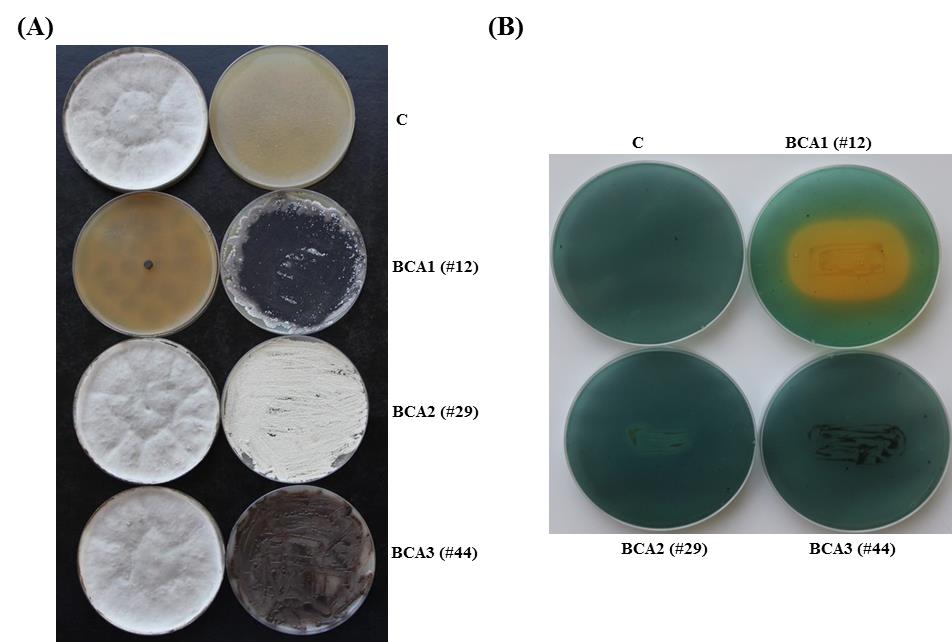
**

**Figure S2.** Effect of volatile antifungal compounds of BCAs on *Lasiodiplodia theobromae* and production of siderophore by BCA candidates. (A) Inhibition of *L. theobromae* mycelial growth by the volatile antifungal compound-producing isolate *Streptomyces samsunensis* UAE1 (BCA1; isolate #12) compared to the non-volatile antifungal compounds producing isolate *S. cavourensis* UAE1 (BCA2; isolate #29) and *Micromonospora tulbaghiae* UAE1 (BCA3; isolate #44). (B) Siderophore production by isolate #12 compared to the siderophore non-producing isolates #29 and #44 on chrome azurol S agar plates. In (A), from upper to lower panel, fish meal extract agar plates (right) are either colonized by no BCA (C), or isolates #12, #29 or #44. In (B), the yellow halo surrounding the colony indicates the excretion of siderophore.

**
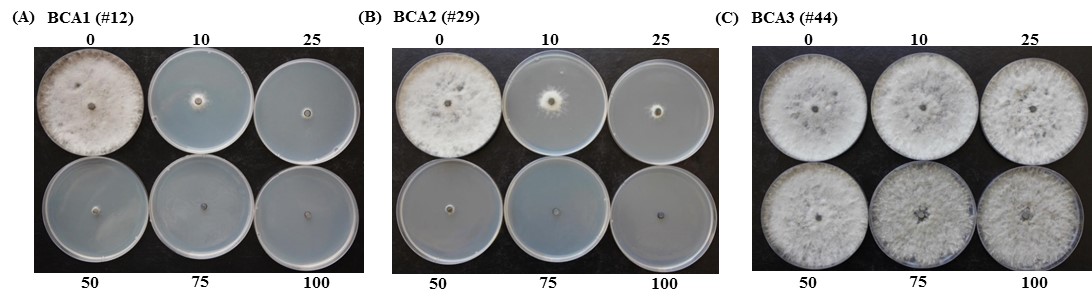
**

**Figure S3.** Effect of filter-sterilized crude culture filtrates of BCA candidates on *Lasiodiplodia theobromae*. Gradual inhibition of *L. theobromae* colony growth on PDA plates containing different proportions (%) of crude culture filtrate obtained from fish meal extract broth only with the antifungal metabolite-producing isolates **(A)** *Streptomyces samsunensis* UAE1 (BCA1; isolate #12) and **(B)** *S. cavourensis* UAE1 (BCA2; isolate #29) compared to **(C)** the non-diffusible antifungal metabolite-producing isolate *Micromonospora tulbaghiae* UAE1 (BCA3; isolate # 44).

**
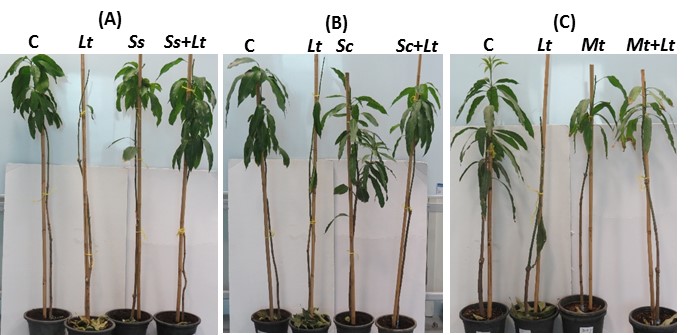
**

**Figure S4.** Antagonistic effect of BCA candidates against mango dieback disease caused by *Lasiodiplodia theobromae* in the greenhouse. Effect of preventive biocontrol treatment of (A) *Streptomyces samsunensis* UAE1 (BCA1; isolate #12); (B) *S. cavourensis* UAE1 (BCA2; isolate #29); (C) *Micromonospora tulbaghiae* UAE1 (BCA3; isolate #44) on mango seedlings at 9 wpi with *L. theobromae*. C, non-inoculated control seedlings, *Lt*, seedlings inoculated with *L. theobromae* only; *Ss*, *Sc* or *Mt,* seedlings inoculated with only *S. samsunensis*, *S. cavourensis* or *M. tulbaghiae*, respectively; *Ss*+*Lt*, *Sc*+*Lt* or *Mt*+*Lt*, seedlings inoculated with the individual BCA, *S. samsunensis, S. cavourensis* or *M. tulbaghiae*, respectively, one week prior to *L. theobromae* inoculation. wpi, weeks post inoculation.

**Table S1. Comparison of morphological, cultural and phenotypic characteristics that distinguish BCA1 (isolate #12) from very closely related species *Streptomyces samsunensis* and *S. malaysiensis.***

| **Characteristics** | **Isolate #12** | ***S. samsunensis*** | ***S. malaysiensis*** |
| --- | --- | --- | --- |
| **Morphological** | | | |
| Morphology of the spore chains | Closed spiral | Closed spiral | Closed spiral |
| Spore surface | Wrinkled (Rugose) | Wrinkled (Rugose) | Wrinkled (Rugose) |
| **Cultural** | | | |
| Color of aerial mycelium on ISP3 | Greyish black | Greyish black | Smoky black |
| Color of substrate mycelium on ISP3 | Greyish yellow | Greyish yellow | Yellow brown |
| Color of diffusible pigment on (ISP3) | None | None | None |
| Production of melanin pigments on peptone-yeast extract-iron agar | - | - | + |
| Production of melanin pigments on tyrosine agar | - | - | + |
| **Phenotypic** | | | |
| Growth on sole carbon sources (%, w/v): | | | |
| Adonitol (1.0) | - | - | + |
| L-Arabinose (1.0) | + | + | + |
| Dextrin (1.0) | + | + | + |
| Lactose (1.0) | + | + | - |
| Salicin (1.0) | - | - | + |
| *myo*-inositol (1.0) | - | - | + |
| Raffinose (1.0) | - | - | + |
| Xylose (1.0) | - | - | + |
| Sodium propionate (0.1) | - | - | + |
| Growth on sole nitrogen sources (0.1 %, w/v): | | | |
| L- Methionine | + | + | - |
| L-Serine | + | + | + |
| Degradation tests (%, w/v): | | | |
| Aesculin hydrolysis (0.1) | - | - | + |
| Arbutin hydrolysis (0.1) | + | + | - |
| Gelatin hydrolysis (0.4) | - | - | + |
| Hypoxanthine hydrolysis (0.4) | - | - | + |
| L-Tyrosine (0.4) | - | - | + |
| Tween 80 (1.0) | + | + | + |
| **Reference** | This study | Sazak *et al.,* 2011 | Al-Tai *et al.,* 1999 |
| +, growth or positive reaction; -, no growth or negative reaction. | | |  |

**Table S2. Comparison of morphological, cultural and phenotypic characteristics that distinguish BCA2 (isolate #29) from very closely related species *Streptomyces cavourensis* and *S. albolongus*.**

| **Characteristics** | **Isolate #29** | ***S. cavourensis*** | ***S. albolongus*** |
| --- | --- | --- | --- |
| **Morphological** | | | |
| Morphology of the spore chains | Straight to flexuous (Rectus-flexibilis) | Straight to flexuous (Rectus-flexibilis) | Straight to flexuous (Rectus-flexibilis) |
| Spore surface | Smooth | Smooth | Smooth |
| **Cultural** | | | |
| Color of aerial mycelium on ISP3 | Yellow | Yellow | White to pale beige |
| Color of substrate mycelium on ISP3 | Yellow-brown | Yellow-brown | Yellow-brown |
| Color of diffusible pigment on (ISP3) | Yellow-brown | Yellow-brown | None |
| Production of melanin pigments on peptone-yeast extract-iron agar | + | + | + |
| Production of melanin pigments on tyrosine agar | + | + | + |
| **Phenotypic** | | | |
| Growth on sole carbon sources (%, w/v): | | | |
| Glucose (1.0) | + | + | + |
| L-Arabinose (1.0) | - | - | + |
| Fructose (1.0) | + | + | - |
| Rhamnose (1.0) | - | - | - |
| Mannitol (1.0) | + | + | - |
| *myo*-inositol (1.0) | - | - | - |
| Raffinose (1.0) | - | - | + |
| Xylose (1.0) | + | + | + |
| **Reference** | This study | Giolotti, 1958 (in Waksman, 1961) | Tsukiura *at al.,* 1964 |
| +, growth or positive reaction; -, no growth or negative reaction. | | | |

**Table S3. Disease severity index (DSI) of *Lasiodiplodia theobromae* inoculated-mango seedlings (cv. Badami) inoculated with each BCA candidate at 3 and 9 wpi (*n* = 6).**

| **Treatment** | **DSI *^a^*** | |
| --- | --- | --- |
|  | **3 wpi** | **9 wpi** |
| C | 0.00 *a* | 0.00 *a* |
| *Lt* | 2.83 *b* | 4.33 *b* |
| *Ss* | 0.00 *a* | 0.00 *a* |
| *Ss+Lt* | 0.17 *a* | 0.17 *a* |
| *Sc* | 0.00 *a* | 0.00 *a* |
| *Sc+Lt* | 0.17 *a* | 0.17 *a* |
| *Mt* | 0.00 *a* | 0.00 *a* |
| *Mt+Lt* | 0.17 *a* | 0.17 *a* |
| *^a^* DSI is on a scale of 5: 0=no infection, 1=1-10%, 2=11-25%, 3=26-50%, 4=51-75%, and 5=76-100% damage (necrotic or dark brown area around the point of inoculation). Values with similar letters are not significantly different at *P* = 0.05.  C, control (no inoculation or treatment); *Lt*, seedlings inoculated with *L. theobromae* only; *Ss, Sc* or *Mt*, seedlings inoculated with a particular BCA. *Ss+Lt*, *Sc+Lt* or *Mt+Lt*, seedlings inoculated with a particular BCA one week before *L. theobromae* inoculation; *Ss*, *Streptomyces samsunensis* UAE1; *Sc*, *S. cavourensis* UAE1; *Mt*, *M. tulbaghiae* UAE1. wpi, weeks post inoculation. | | |
